# Probing the Solubility of Imine-Based Covalent Adaptable Networks

**DOI:** 10.1021/acsapm.3c01472

**Published:** 2023-12-19

**Authors:** Sybren Klaas Schoustra, Vahid Asadi, Maarten Marinus Johannes Smulders

**Affiliations:** Laboratory of Organic Chemistry, Wageningen University, Stippeneng 4, 6708 WE Wageningen, The Netherlands

**Keywords:** covalent adaptable networks, dynamic covalent chemistry, polymers, solubility, imines, polymer recycling

## Abstract

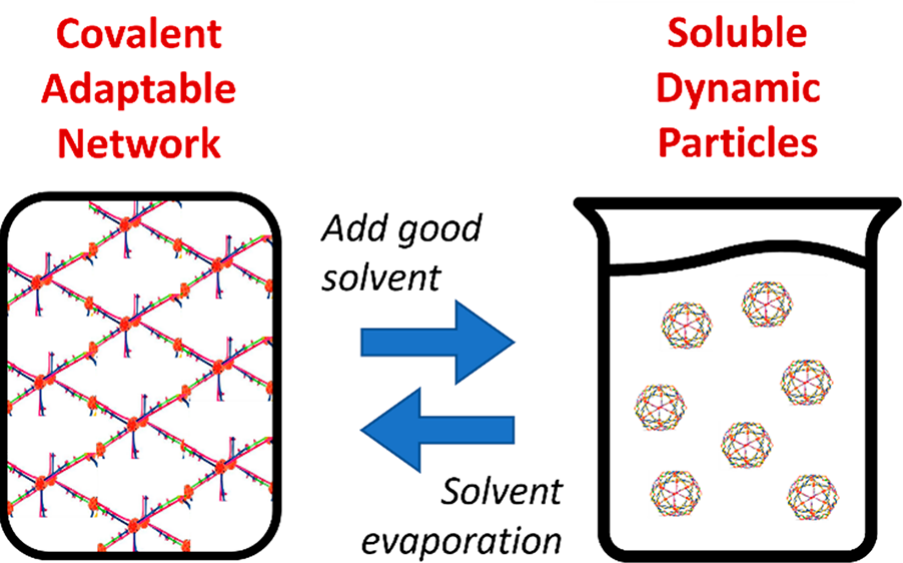

Covalent adaptable networks (CANs) are polymer materials that are covalently cross-linked via dynamic covalent bonds. The cross-linked polymer network is generally expected to be insoluble, as is seen for traditional thermosets. However, in recent years, it has become apparent that—under certain conditions—both dissociative and associative CANs can be dissolved in a good solvent. For some applications (e.g., those that require long-term (chemical) stability), the solubility of CANs can be problematic. However, many forget that (selective) solubility of CANs can also be applied advantageously, for example, in recycling or modification of the materials. In this work, we provide results and insights related to the tunable solubility of imine-based CANs. We observed that selected CANs could be fully dissolved in a good solvent without observing dissociation of imines. Only in an acidic environment (partial) dissociation of imines was observed, which could be reverted to the associated state by addition of a base. By adjusting the network composition, we were able to either facilitate or hamper solubility as well as control the size of the dissolved particles. DLS showed that the size of dissolved polymer particles decreased at lower concentrations. Similarly, decreasing cross-linking density resulted in smaller particles. Last, we showed that we could use the solubility of the CANs as a means for chemical recycling and postpolymerization modification. The combination of our studies with existing literature provides a better understanding of the solubility of CANs and their applications as recyclable thermosets.

## Introduction

Thermosets are widely applied materials as their covalently cross-linked polymer structure enables superior strength and toughness compared to linear or branched thermoplastics. The permanent network structure of thermosets, however, comes with the issue that recycling or reprocessing is impossible. Once the polymer network has been set, it is permanent. A solution to this problem was presented by the development of covalent adaptable networks (CANs).^1,2^ CANs are thermosets by nature, as they have the same covalently cross-linked network structure, although with the exception that they contain dynamic covalent bonds. These dynamic covalent bonds can perform bond exchange reactions,^3^ which enable polymer chains within the network to be exchanged, allowing flow and stress relaxation within the material.^4,5^ To activate this process, bond exchange requires some sort of activation or stimulus. This can, for example, be achieved by heating and/or with a catalyst,^6^ which can even be internal.^7,8^

A breakthrough for these dynamic polymers was when in 2011 Leibler and co-workers documented on dynamic polymer networks that showed Arrhenius-type behavior in their temperature dependence of the viscosity, similar to vitreous silica. They, therefore, coined the term “vitrimer”,^4^ which is now commonly used in the literature. Since then, many researchers have expanded the field, and different types of dynamic covalent bonds have been studied for their inclusion in CANs.^9^

The covalently cross-linked network structure of thermosets is what generally protects them from the influence of solvents. Many thermosets are still able to swell in good solvents, but fully dissolving them is inherently impossible. Current efforts have, however, been made regarding controlled degradation of thermosets by means of solvolysis.^10^

For CANs, the effects of solvent resistance, swelling, and solubility are not as trivial. First, CANs tend to swell more than classical thermosets, as bond exchange and cleavage can take place during swelling.^11−13^ Here, the mechanism of the bond exchange reaction is crucial. In general, we can classify the bond exchange reaction to be either dissociative or associative.^14,15^ For the dissociative exchange mechanism, a bond is first broken before a new bond is formed, leading to a temporary decrease in cross-linking density. For the associative mechanism, a new bond is formed before the old bond breaks, leading to a temporary increase in cross-linking. From this perspective, it could be postulated that the dissociative mechanism would increase the possibility to dissolve respective CANs,^16^ as the network could be broken down back to soluble monomers or oligomers. Meanwhile, this would not be the case for CANs relying on associative bond exchange, as the network would never break down.^15^ As such, associative CANs (vitrimers) were initially expected to never fully dissolve in any solvent.^17^ However, there has recently been debate on the solubility of both dissociative and associative CAN.^18−21^

**Figure 1:**
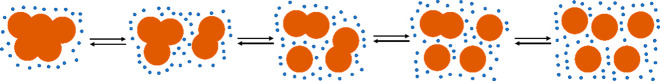
Schematic representation of the continued merging and separation of dynamic polymer clusters (pictured in orange) facilitated by the solvent (pictured as blue dots). Note that both merging and separation proceed via dynamic covalent bond exchange reactions.

With a theoretical “patchy particle model”, Smallenburg, Leibler, and Sciortino were able to further speculate on the swelling behavior and dissolution of vitrimers.^22^ For typical soluble materials, the addition of a good solvent favors the formation of a dilute phase consisting of small clusters. However, the calculated phase diagram of Smallenburg, Leibler, and Sciortino demonstrated that vitrimers would never fully dissolve,^22^ as was initially proposed on the basis of experimental results.^4^ They found that only monomers and smaller clusters could escape from the network upon dilution, while the majority of the bulk network remained as a whole, emphasizing that separation into the dissolved or nondissolved state is driven purely by entropy.^22^ Here, it is important to note that bond exchange reactions enable small clusters to separate from the bulk, while small clusters may also assemble and recombine via bond exchange with other clusters (Figure 1). In theory, this can be seen as an equilibrium reaction.

Simulations with the patchy particle model showed that assembly of smaller particles to a larger aggregate is thermodynamically favorable,^22^ which is in favor of the postulate against full solubility of vitrimers. However, although the theoretical model shows good coherence to the description of vitrimer-like behavior,^4,6^ some questions and discussions regarding the full extent of swelling and solubility remain.^12,23^ Some understudied parameters are, for example, the type of solvent (e.g., polar/apolar, protic/aprotic), the cross-linking density of the polymer network, the concentration of dynamic covalent bonds, or the exchange kinetics of the bond exchange reactions. Nicolaÿ and co-workers, for example, observed that polybutadiene vitrimers based on dioxaborolane chemistry were soluble in THF after prolonged immersion time at room temperature, which they related to the low molar mass of the thermoplastic precursor, the low number of cross-links, and the dynamics of the dioxaborolane exchange.^11^

Another discussion focuses on the size (distribution) of the particles in the solvent, and questions whether to call solvent–polymer mixtures either a solution, colloid, or suspension. Typically, a mixture is considered a solution when the dissolved particles are smaller than 1 nm, a colloid with sizes between 1–1000 nm, and a suspension when over 1000 nm.^24^ For this reason, true solutions do not scatter light, as the particles are too small, whereas colloids do scatter light. The scattering of visible light by colloidal particles is also known as the Tyndall effect. Note, however, that transparency of the mixture does not always mean full dissolution.^25,26^ For consistency considerations, in this work, we will refer to our solvent–polymer mixtures as “solutions”, unless explicitly stated otherwise.

**Figure 2:**
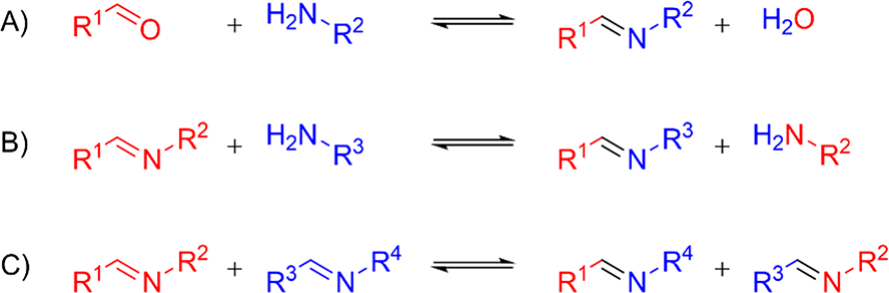
Overview of bond exchange reactions of imines: (A) condensation and hydrolysis of aldehyde and amine, (B) transimination of imine and amine, and (C) imine metathesis between different imines.

Among the different types of CANs, polyimines are well-studied examples,^27−29^ which we have also extensively studied in our previous works.^30−33^ An interesting feature of imines is that they can perform both associative and dissociative bond exchange.^34−36^ Three ways of imine exchange are considered: hydrolysis, transimination, and metathesis (Figure 2). The hydrolysis (and reformation via condensation) has a dissociative mechanism (Figure 2A), whereas the transimination (Figure 2B) and metathesis (Figure 2C) are considered associative. The underlying mechanisms of the imine exchange have been studied thoroughly for a long time.^37−41^ However, a full understanding, especially regarding the metathesis reaction,^42,43^ is still a topic of discussion and may require further investigations.^35^

Even though imines are able to perform bond exchange via both dissociative and associative mechanisms, methods have been developed to push the main exchange path to either of the two. For example, increasing the stability of the imine (e.g., via aromatic conjugation) can suppress hydrolysis.^31,44^ Conversely, acidic aqueous environments can stimulate dissociative exchange via hydrolysis and condensation.^45^ When an excess of amines is used during the synthesis, and free available amine groups remain present in the polymer network, the transimination reaction can be promoted.^34,36^ When using a stoichiometric amount of aldehyde and amine, and all amines are converted to imines, the metathesis reaction will become the main mode of bond exchange.^31,46^

The type of bond exchange of imines is important for the consideration of the solubility of polyimine CANs. First, aqueous acidic environments are known to promote the hydrolysis of imines, leading to depolymerization into soluble particles or monomers.^29^ Second, by means of solvent-assisted solubility, primary amines can be used as the solvent, which can perform transimination with the polymer to split it in parts.^47^ More importantly, however, it was noticed that specific polyimines showed (partial) solubility in organic solvents without the addition of either an acid or primary amines.^12,48,49^ Based on these initial observations, speculations were made that fast imine exchange could facilitate rearrangements of the polymer network into smaller soluble particles.^49^ However, to fully understand and probe the solubility of imine-based CANs, more research is required.

In this work, we investigate several factors that affect the solubility of imine-based CANs. We also look into the materials in their dissolved state to get insights into what is happening on the microscopic level. In our studies, we included the selection of several common but chemically different organic solvents. We then varied the composition of the imine network and observed distinct relations between the chemical structure of the polymers and their solubility and solvent resistance for specific solvents. By using NMR and DLS, we were also able to show that the polyimine networks likely rearrange into smaller soluble nanoparticles for which the size was affected by the concentration and the composition of the polymers. Last, we showed that dissolution of the polyimine CANs could be applied as a means for chemical recycling of the materials. In order to place our observations from polyimine CANs in a broader perspective for other CANs, we also prepared vinylogous urea (V-Urea) CANs from similar compositions as the polyimines and compared their corresponding dissolution behavior.

## Results and Discussion

### Synthesis of Polyimine CANs

**Figure 3:**
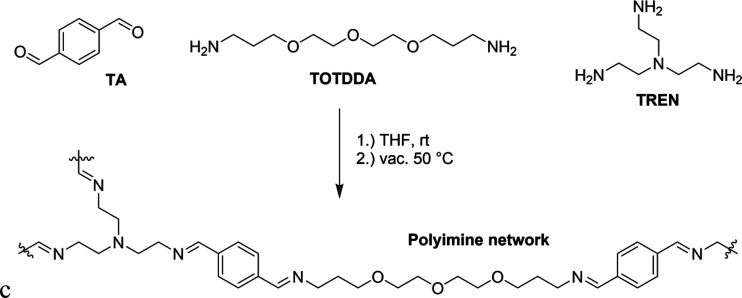
Reaction scheme for the synthesis of polyimine CANs from TA, TOTDDA, and TREN. For PI-30, a stoichiometric amount of aldehyde and amine groups was used, for which 30% of amine groups link to TREN and 70% to TOTDDA.

To study the solubility of polyimine CANs, we started with the preparation of a polymer network from terephthalaldehyde (TA), 4,7,10-trioxa-1,13-tridecanediamine (TOTDDA), and tris(2-aminoethyl)amine (TREN) (Figure 3). A stoichiometric amount of aldehyde to amine groups was used, where 30% of amines were from TREN and 70% from TOTDDA; hence, the abbreviation PI-30 was used. The synthesis was performed according to our previously documented synthesis for polyimine CANs,^31^ in which the monomers were mixed in a small amount of THF and were then poured into a glass Petri dish. They were left at room temperature and open to the air overnight, during which most of the solvent evaporated. To remove any remaining solvent and water from the polymer films, they were placed in a vacuum oven at 50 °C for at least 24 h. Once fully dried, the materials were used for analysis. If needed, they could be hot-pressed at 100 °C into a desired shape. FT-IR was used to check for full conversion by the disappearance of the aldehyde signal (1686 cm^–1^) and the appearance of the imine signal (1641 cm^–1^). The materials appeared as rubbery transparent orange films, for which a *T*_g_ of −14 °C was determined with DSC and a rubbery plateau modulus of 0.5 MPa was determined with rheology. See the Supporting Information for additional details on the synthetic procedure and analysis.

### Solubility of Polyimine CANs

Normally, the initial exposure of a cross-linked thermoset material to a good solvent would only result in a small soluble fraction containing either unreacted monomers and/or small fragments or chains that were not connected to the rest of the network structure (e.g., small loops or terminated oligomers). However, the main polymer network would not dissolve and only swell to some degree. This swelling is the result of solvent molecules that penetrate into the polymer network, causing the network to be stretched outward.^13^ The permanently cross-linked structure of thermosets, however, prevents them from being ruptured.^50^ For CANs, while the network is under stress, bond exchange reactions cause stress relaxation, which effectively makes the network more stretchable. Additionally, rearrangements of polymer chains can cause the formation of small loops or loose particles that separate from the network. These smaller particles can then dissolve in the solvent.

**Figure 4:**
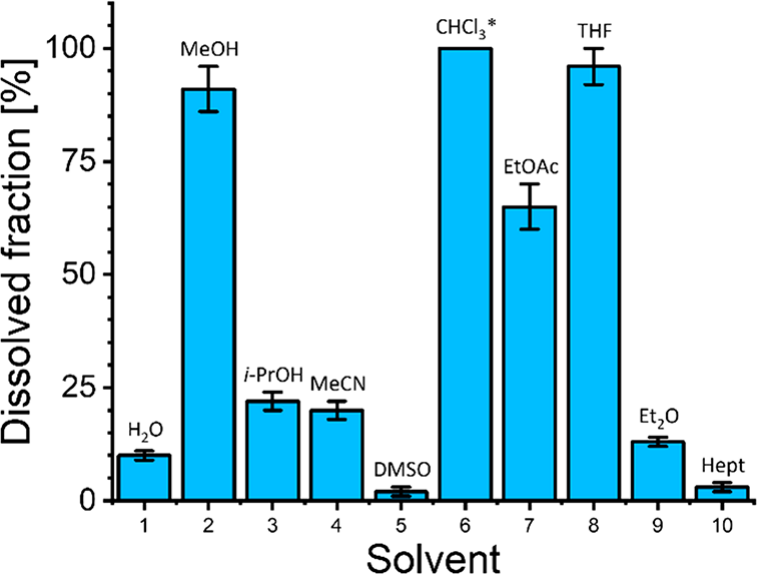
Dissolved fractions of PI-30 in several common solvents, determined by the addition of 100 mg of polymer material to 10 mL of the respective solvent (performed *in triplo*). Only for CHCl_3_ full solubility (100%) was observed. As such, it does not contain an error margin (indicated by the ∗). Solvents were ordered from the most polar (left) to apolar (right).

To determine the solubility of the prepared polyimine material, we selected several organic solvents and placed 100 mg of polymer in 10 mL of the respective solvent. The vials were then capped and left for 10 days at room temperature. Afterward, the liquid and solid phases were separated and dried to determine the dissolved and nondissolved fractions. The dissolved fractions in each solvent are shown in Figure 4 and are ordered from most polar (left) to apolar (right).

Most solvents offered relatively poor solubility of the polyimine material, and few showed reasonable solubility. Chloroform was the only solvent able to fully dissolve all material. MeOH, THF, and EtOAc still offered reasonably high solubility (>50%). Interestingly, the solubility did not seem to correlate to the solvent polarity or dielectric constant (see the Supporting Information, Figures S12 and S13), nor was there a clear trend between protic and aprotic solvents. This was rather unexpected, as other studies did describe relations between imine exchange and solvent properties^34,46^ as well as network polarity.^32^ The dynamics of the bond exchange alone might therefore not directly correlate to better solubility. Instead, the network structure may also play a larger part here, as the nature of the network may affect the penetration of specific solvents.^51^ It is thus likely that an interplay between the bond exchange kinetics and network integrity may operate concurrently.

It should also be noted that long soaking times were required to dissolve all material. Even in chloroform, several hours were required before full solubility was observed. Typically, we observed that the polymers first underwent swelling, and only afterward did the actual dissolution process start, rather than the material being broken down from the outside inward. This suggests that the penetration of solvent molecules into the polymer network is a slow process and likely is one of the rate-limiting steps in the dissolution process.

**Figure 5:**
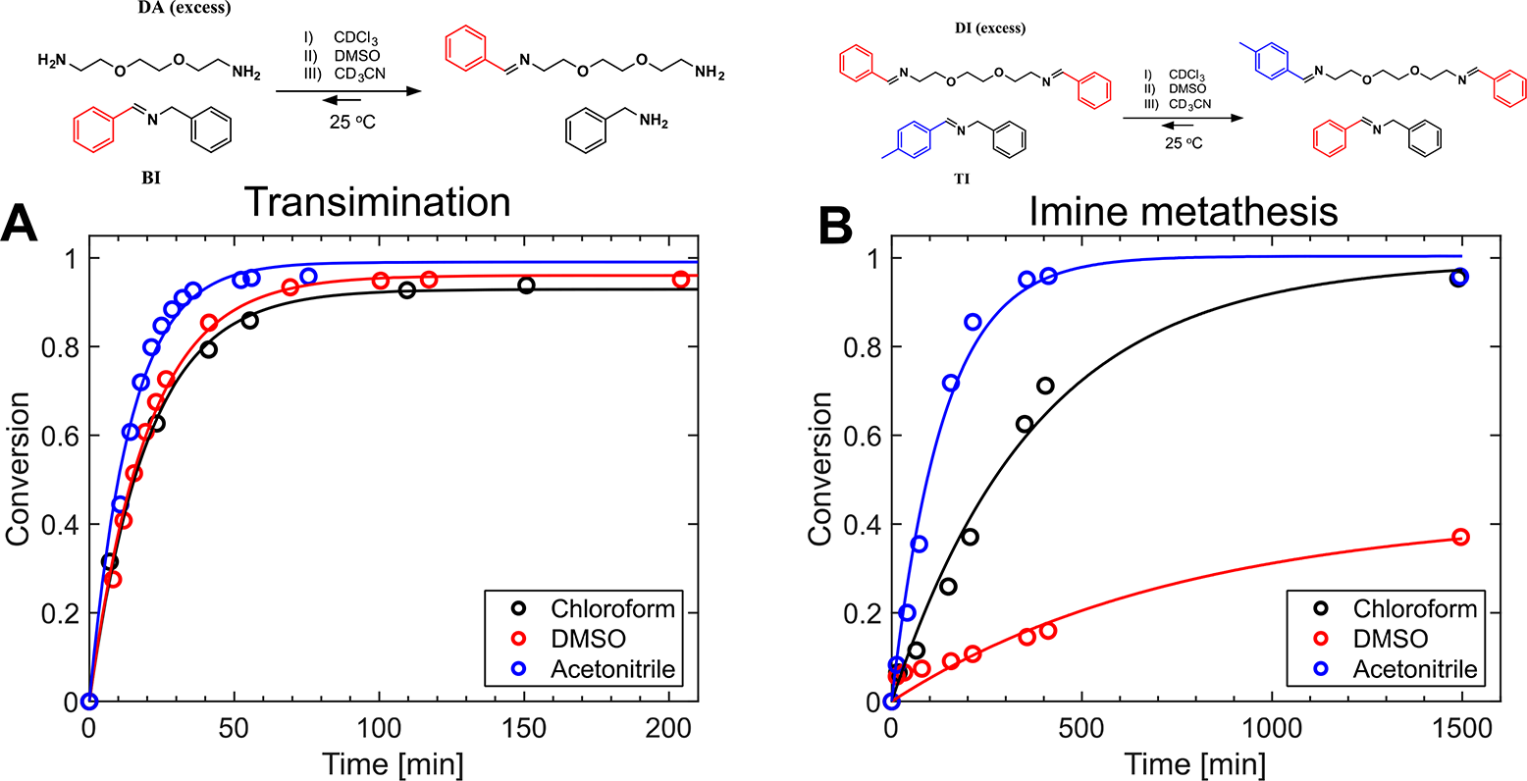
Imine bond exchange kinetic study in chloroform, DMSO, and acetonitrile. (A) Transimination exchange reaction in the presence of excess diamine. (B) Imine metathesis exchange reaction in the presence of excess diimine.

To evaluate this hypothesis of interplay between bond exchange kinetics and network integrity, we studied the imine bond exchange reactions (transimination and metathesis) in three different solvents (chloroform, acetonitrile, and DMSO), which show different solubility toward polyimine CANs (and in which the molecules are soluble). Figure 5 shows the scheme of studied reactions and their conversion over time in different solvents, which then were fitted with the first-order reaction model (*y* = *A*(1 – e^–*kt*^)) to calculate the rate constants (*k*), as summarized in Table 1. All of the details of this kinetic study can be found in the Supporting Information. Based on these kinetic studies, the transimination reaction was found to occur with similar rate constants in chloroform and DMSO; however, it showed a higher rate constant for acetonitrile. However, the metathesis exchange is the more relevant exchange type during the dissolution, as the polyimine CANs in this study have been synthesized with an aldehyde:amine ratio of 1:1. In the case of imine metathesis, the exchange occurred noticeably faster in acetonitrile than chloroform while it was slow in DMSO. As opposed to being the most favored solvent in terms of exchange rate, acetonitrile is not the best in the solubility of the polyimine sample. Therefore, the molecular exchange kinetics are not the only definitive factor here. To investigate how fast these three solvents can penetrate and swell the sample, we monitored the dissolution process over time, as shown in Figure S16. We defined a characteristic dissolution onset time where the sample has lost 5% of its weight (*t*_0_) during the dissolution process, and it is listed in Table 1. The characteristic times are 0.1, 3.75, and 19 h for chloroform, acetonitrile, and DMSO, respectively, showing that even though acetonitrile provides a fast exchange reaction rate it has slow penetration into the sample, leading to lower solubility than chloroform. These results support our hypothesis of an interplay between exchange kinetics and solvent penetration. Additional dissolution experiments were also performed using anhydrous methanol, as well as neutral and anhydrous chloroform to rule out the potential effect of trace amounts of water or acid (HCl in chloroform), shown in Figure S17.

**Table 1:** Imine Exchange Reaction Rate Constants and Dissolution Onset Time in Different Solvents

solvent	*k*_transimination_ (min^–1^)	*k*_imine metathesis_ (min^–1^)	*t*_0_ (h)
chloroform	4.98 × 10^–2^	2.61 × 10^–3^	0.1
DMSO	5.01 × 10^–2^	1.30 × 10^–3^	19
acetonitrile	7.03 × 10^–2^	7.39 × 10^–3^	3.75

**Figure 6:**
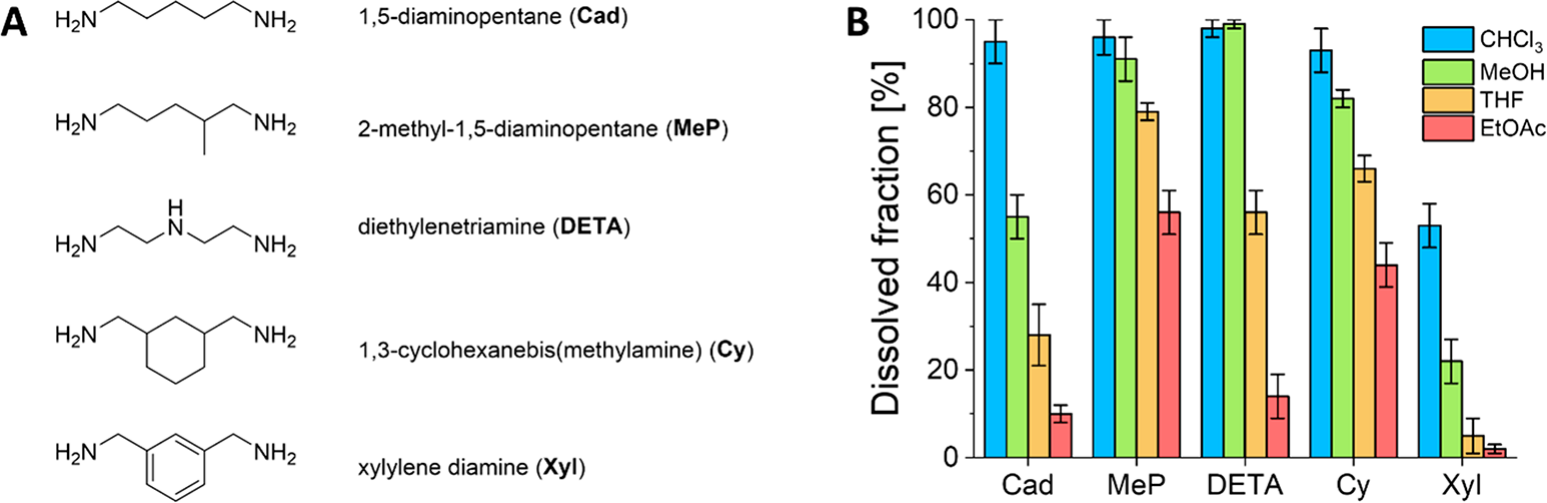
(A) Chemical structures of the different diamine monomers, including full name and abbreviation. (B) Dissolved fractions of the polyimine materials synthesized from each of the pictured diamines (together with TA and TREN, with a cross-linking of 30%).

Next, five additional polyimine materials were prepared with diamines of similar length but different chemical nature (Figure 6A). The aldehyde (TA) and triamine (TREN) monomers were held constant for all materials. We hypothesized that chemical differences in the chains of the network structure would affect the solubility of the polyimine materials. The mechanical properties of these five polyimine materials can be found in the Supporting Information (Figure S18). With the chosen variations, we envisioned to gain a better understanding of which chemical groups would facilitate better solubility or solvent resistance. For example, compared to linear 1,5-diaminopentane (Cad), adding a methyl branch (MeP) or incorporating a cyclohexane ring (Cy) could affect the polymer chain alignment and flexibility. Additionally, incorporation of an aromatic benzene ring (Xyl) was expected to affect the network integrity.^52^ Last, diethylenetriamine (DETA) was expected to potentially affect the imine kinetics as a result of the polarity of the chain and the potential to form hydrogen bonds with the imines.^28,32^

A similar solubility test as before was performed for all five polyimine materials, using chloroform, MeOH, THF, and EtOAc as solvents. From the results (Figure 6B) some clear conclusions could be drawn by relating solubility to the chemical structure of the diamine chains. First, it was observed that the xylylene (Xyl) materials showed significantly higher solvent resistance than any of the other materials for each of the tested solvents. In addition, Xyl exhibited a higher modulus compared to other samples in the rubbery region, as evidenced by the frequency sweeps measurements at 130 °C (Figure S18). This can be expected, as xylylene groups have been applied in other materials to create tougher networks compared to materials made with simpler linear amines.^52^ A possible explanation for this might be that π–π stacking of the aromatic rings forms additional (weak) supramolecular cross-links in the polymer network.^53^ Next, by comparing the Cad and MeP materials, we observed that branching of the diamine structure significantly improved the solubility of the materials for all tested solvents. In addition, a decrease in the modulus (both in the glassy and rubbery region) and also in the *T*_g_ (determined from the peak of tan(δ) in temperature sweep measurements) was also observed for MeP compared to Cad, indicating a more flexible network and a lower cross-link density. This observation could be explained as branching of polymer chains generally results in a more flexible network and loss of crystallinity,^54^ combined with the lower cross-linking density, which could facilitate better penetration of solvent molecules into the network. A similar effect was observed for the Cy material, as the cyclohexane structure results in more amorphous materials, as shown by a lower plateau modulus and hence less cross-linking density compared to the Cad material. Last, we noticed significantly improved solubility of DETA material in methanol, which is likely related to the hydrogen-bonding potential of the secondary amine groups with the solvent,^28^ although the effect of the secondary amine on the imine exchange kinetics is also expected to play a role here.^32^

Given the observations from the solubility tests (Figure 6), some hypotheses can be made regarding the mechanisms involved in the dissolution. We observed that penetration of solvent molecules into the network is essential for the dissolution. However, it remains challenging to study what happens once the material is in its swollen state. When solvent molecules penetrate the network structure, the network is being stretched outward, first resulting in a swollen state.^55^ In order to compensate this outward force, bond exchange reactions could cause the polymer chains to rearrange, similar to stress–relaxation mechanisms. These rearrangements could, in turn, cause rupture of the (small) parts of the network. Once these small parts are released from the network, they can diffuse into the solvent. Over time, when more of these small particles separate from the bulk into the solvent, the material is essentially being dissolved. The exchange of polymer chains can, in theory, proceed via associative exchange. However, in a (very) short frame in time, the imines could potentially also dissociate into aldehyde and amine (given that water is present in the system) and immediately react again at a different location. This could, however, not be observed with, e.g., NMR, as the time interval in which this mechanism occurs would be extremely short.

### Solubility of V-Urea CANs

**Figure 7:**
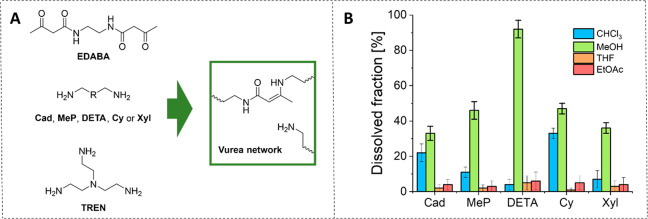
(A) Synthetic overview for the preparation of V-Urea networks from EDABA, TREN, and a variable diamine monomer (see Figure 6 for the structure of all diamines). (B) Dissolved fractions for the different V-Urea networks in several common organic solvents, determined by soaking 100 mg of polymer into 10 mL of the solvent.

To transfer the observations made for our polyimine CANs to other CANs, a similar solubility experiment was performed for vinylogous urea (V-Urea) networks. V-Urea networks have a synthetic design similar to that of imines, but the aldehyde is replaced by an acetoamide. The V-Urea networks perform bond exchange via transamination, which occurs via an associative mechanism.^56^ Note, however, that for V-Ureas an excess of amine is required to facilitate the transimination reaction, which is not the case for imines, as polyimines are generally synthesized from stoichiometric amounts of aldehyde and amine. To allow a proper comparison, for the synthesis of V-Urea networks, the same amine monomers were used as for the imine networks but with a 10 mol % excess of amine groups. They were then reacted with ethylenediamine-*N*,*N*′-bis(acetamide) (EDABA) to construct V-Urea networks (Figure 7A). The synthetic procedure was similar to that for the polyimines, except that DMF was used as the solvent, and a temperature of 80 °C was required (see the Supporting Information for further experimental details). Formation of the V-Urea materials was confirmed by FT-IR, as the C=O stretch signal of the acetamide ketone around 1700 cm^–1^ fully disappeared, in line with earlier reported observations.^56^

After successful synthesis of the V-Urea networks, they were soaked in either chloroform, MeOH, THF, or EtOAc, using the same procedure as before for the polyimine CANs. The dissolved fractions were again determined (Figure 7B). We observed that in general the V-Urea networks had greater solvent resistance than the imines. We did, however, clearly see that dissolution is observed in MeOH. Particularly the V-Urea networks with DETA as diamine showed very good solubility (∼90% dissolved fraction). By adding double the amount of solvent (20 mL of solvent to 100 mg of polymer), all material eventually dissolved. In chloroform, we also observed some solubility for Cad and Cy V-Ureas, but all other tested solvents and materials showed high solvent resistance (<10% dissolved fractions). Likely, the hydrogen-bonding potential of MeOH might facilitate better solubility of the V-Urea networks, whereas for the other nonprotic solvents this is lacking. Specifically for DETA, the extra secondary amine in the chain facilitates even more hydrogen-bonding potential with the solvent, resulting in enhanced solubility, as was also seen for the polyimines. Apart from the network effects, the hydrogen-bonding potential of MeOH with V-Ureas might also cause enhanced bond exchange, resulting in better solubility.

In short, although we do observe generally good solvent resistance of the associative V-Urea CANs, by choosing a specific solvent (this time MeOH), we can facilitate the solubility of the material, which can be further enhanced by the choice of the diamine. As such, when a CAN exchanges via an associative mechanism, this does not automatically imply that the CAN is insoluble. And although the mechanism of the bond exchange may play an important role in the possibility of dissolving a CAN, the nature of the polymer network also significantly affects the solubility. In this regard, it is of interest to point out that for the V-Urea CANs the exchange reaction can be frozen by removing the excess amines inside the materials, making it possible to decouple the effect of exchange kinetics and swelling in the dissolution process.

### NMR Analysis of Dissolved Polyimines

**Figure 8:**
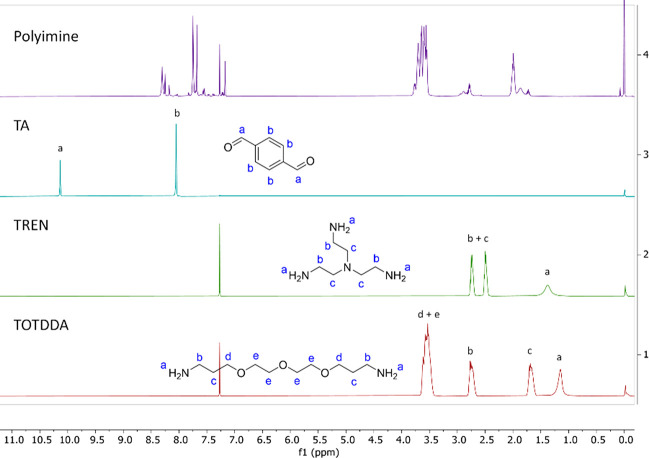
From top to bottom: stacked ^1^H NMR spectra of the dissolved PI-30 (after immersion for 24 h in CDCl_3_) (purple), TA (blue), TREN (green), and TOTDDA (red). All spectra were measured in CDCl_3_ (solvent signal at 7.26 ppm) with TMS as a reference (signal at 0.00 ppm).

When dissolving the polyimine CANs, it was necessary to make sure that we indeed dissolved polymers and not dissociated the polymers back to monomers. For this, NMR analysis was performed. First, we examined the PI-30 material, which was dissolved in CDCl_3_ in an NMR tube. The ^1^H NMR spectra of the dissolved polymers were then analyzed and compared to all individual starting materials (Figure 8). The NMR spectra showed that the imines (8.1–8.4 ppm) stayed intact, and no dissociation back to aldehydes (10.1 ppm) and amines (1.0 ppm to 1.5 ppm) was observed. This observation, in combination with the fact that the network does dissolve, implies that the polymer network reorganizes into small soluble particles such as loops or vesicles,^49,57^ rather than dissociating back to monomers. This is an important result, as it shows that soluble polymeric structures can be formed via bond exchange of the CANs without degrading the material back to monomers. ^1^H NMR spectra of the Cad, MeP, DETA, Cy and Xyl imines were also measured, which all showed that imine groups stayed intact during the dissolution (see the Supporting Information for all NMR spectra).

Dissociation of imines can, however, be achieved by addition of acid.^58^ To test this for the PI-30 material, acetic acid was added to a solution of the dissolved polymer in CDCl_3_. We observed that after the addition of acid partial dissociation of imines occurred (see the Supporting Information). An equilibrium between imine and aldehyde was formed, which shifted more toward the dissociated products when the concentration of acid was increased. Neutralizing the solution by addition of triethylamine, however, was possible to fully push the equilibrium back toward the formation of the imines (see the Supporting Information).

### Size of Dissolved Particles

To further study how the polyimines behaved in solution, dynamic light scattering (DLS) was used to determine the size of the dissolved particles. Three solutions of PI-30 were prepared with concentrations of 0.1, 1.0, and 10 g/L in chloroform. In addition, we synthesized a new polyimine material with a lower cross-link density, for which the TOTDDA monomer was replaced with a longer poly(ethylene glycol) chain with an *M*_n_ ∼ 1500 g/mol. This material was named PEGI-30. After the synthesis of PEGI-30, three solutions were again prepared with concentrations of 0.1, 1.0, and 10 g/L in chloroform. All solutions were kept at room temperature for 4 days before they were analyzed with DLS to make sure that a stable size distribution was obtained.

**Figure 9:**
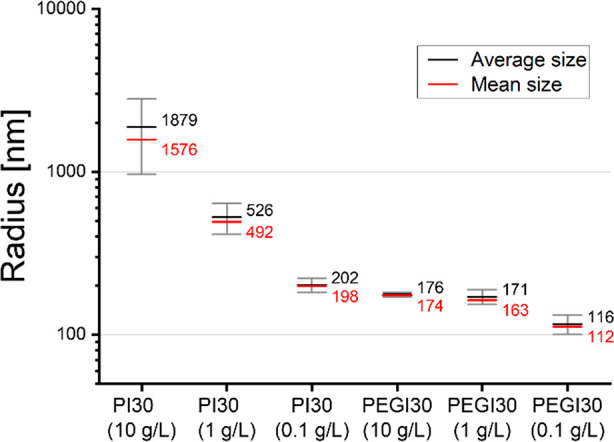
Size of polyimine particles of PI-30 and PEGI-30 for concentration of 10, 1, and 0.1 g/L in chloroform. The average hydrodynamic radius is given in black, and the mean is given in red.

For PI-30, we observed a clear trend in which a lower concentration resulted in smaller particle sizes, up to almost an order of magnitude difference between the 10 and 0.1 g/L (Figure 9). For the PEGI-30 material, the difference between 10 and 1 g/L solutions was relatively small, but at the lowest concentration of 0.1 g/L, the size of the dissolved particles decreased more clearly. The results of smaller particle sizes at lower concentrations are in favor of the hypothesis that when diluting (i.e., ratio of polymer to solvent decreases), the chance of particles meeting and reassociating is smaller. As such, the equilibrium shown in Figure 1 is pushed to the right.

From the DLS results it was also observed that PEGI-30 showed smaller particle sizes compared to PI-30, which is likely the result of the lower cross-linking density. A lower cross-linking density facilitates easier penetration of solvent molecules into the polymer and as such facilitates easier dissociation of large polymer particles into smaller ones.^12,50^ A similar observation was made by Tellers and co-workers, who noticed a trend between the solubility and cross-linking density of vinylogous urethane CANs.^21^ Where highly cross-linked materials showed to be only partially soluble, leaving a low fraction of gel-like residues, the lower cross-linked materials were able to fully dissolve within several hours. It should, however, also be noted that by using longer polymer chains to decrease the cross-linking density, the relative amount of dynamic covalent bonds in the material decreases. As such, fewer bond exchange reactions might take place, affecting the overall dynamic behavior.

**Figure 10:**
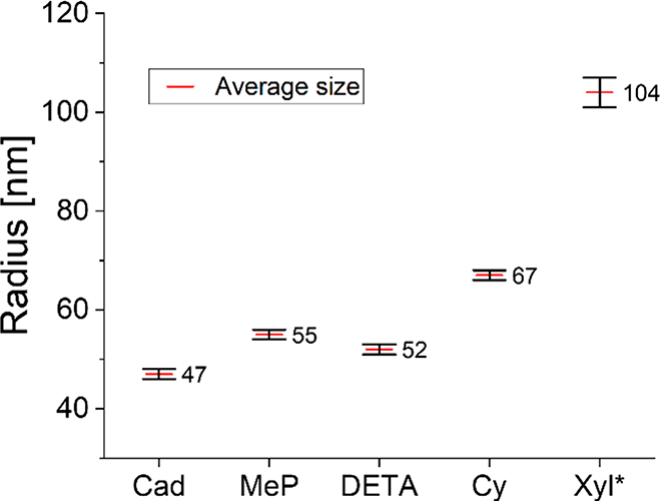
Hydrodynamic radius of dissolved polyimines synthesized from different pentane diamines. All measurements were performed in chloroform at a concentration of 1 g/L. *Because the Xyl material did not fully dissolve and the remaining mass remained on the bottom of the flask, only the dissolved particles were observed.

The same DLS experiment was then also performed for the polyimines presented in Figure 6A, with a concentration of 1 g/L. These results showed that the dissolved particles of Cad, MeP, and DETA had similar sizes with a hydrodynamic radius of around 50 μm (Figure 10). Larger sizes were observed for Cy (67 μm) and Xyl (104 μm). It is likely that the bulkiness of the diamine chain has an effect here, as their chain length is similar but their bulkiness is not: Cad, MeP, and DETA have similar bulkiness of the chains, but Cy and Xyl contain larger cyclohexane and benzene rings, respectively. The variations in particle size might thus not per se be related to the difference in solubility for these specific cases but rather to the bulkiness of the polymer chains. Together with the previously observed results from PI-30 and PEGI-30 (Figure 9), we thus expect that concentration, cross-linking density, and bulkiness of the monomer units are the most important factors that determine the size of the dissolved particles.

### Recycling via Dissolution

An important application for the solubility of CANs can be found in chemical recycling. In earlier work, both dissociative and associative CANs could be recycled via so-called solvent-assisted solubility.^18,59,60^ In solvent-assisted dissolution, a specific solvent is chosen that can perform bond exchange with the dynamic bonds in the polymer. As a result, the polymer network is broken down into smaller, end-capped pieces. To reverse this depolymerization, the volatile solvent can be evaporated again to reform the network structure. Such solvent-assisted dissolution has already been applied to various types of CANs. For example, ester-based CANs could be dissolved using alcohols as solvent,^61,62^ disulfide-based CANs could be dissolved by using thiols as the solvent,^63,64^ and imine-based CANs could be dissolved using primary amines as the solvent.^47,52^ As mentioned before though, this technique of solvent-assisted dissolution does, by definition, not dissolve the actual polymer but rather cuts it into small soluble end-capped pieces or monomers. It could, therefore, perhaps better be described as a practice of degradation or depolymerization.^65^

We were, however, able to fully recycle our polyimine CANs via dissolution without requiring primary amines to break down the polyimine network, but simply by dissolving the CANs in pure THF. For this recycling test, PI-30 materials were synthesized and their material properties were determined using rheology. The materials were then cut into small pieces and fully dissolved in THF. Evaporation of the solvent resulted in the formation of a new recycled polymer film. The newly obtained film was analyzed and compared to the pristine material (see the Supporting Information for further experimental details).

**Figure 11:**
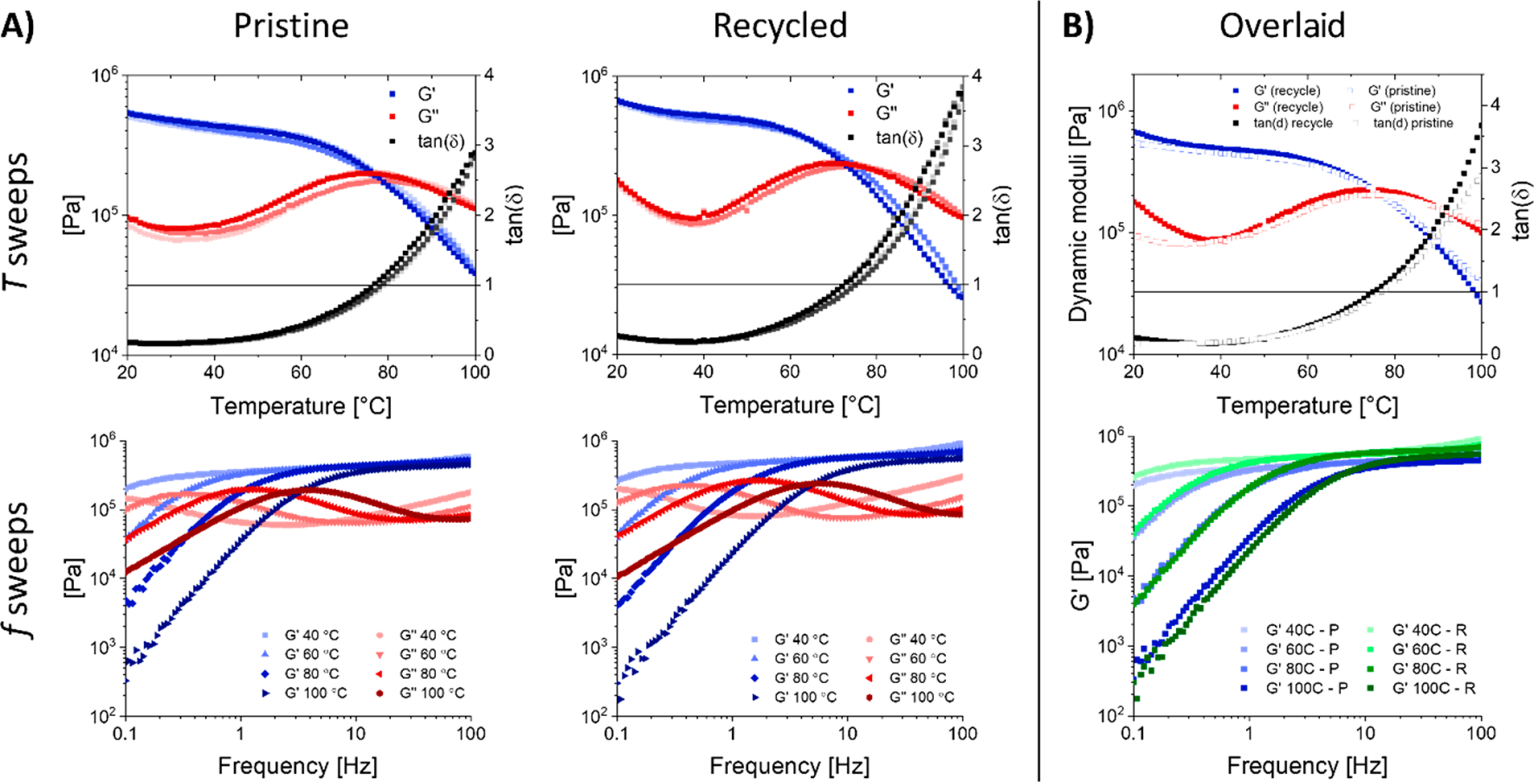
(A) Top: temperature sweeps of pristine (left) and recycled (right) polyimine material. In blue is the storage modulus (*G*′), in red is the loss modulus (*G*″), and in gray is the tan(δ). The *T*_cross_ was determined at tan(δ) = 1. Bottom: frequency sweeps of pristine (left) and recycled (right) polyimine material. In blue is the storage modulus (*G*′) and in red is the loss modulus (*G*″), where a darker shade of the color indicates a higher temperature. (B) Top: overlaid plot of representative temperature sweeps for pristine and recycled material. Bottom: overlaid plot of the *G*′ of representative frequency sweeps for pristine and recycled material.

From temperature sweep experiments (Figure 11), we concluded that the elastic (*G*′) and viscous (*G*″) moduli of pristine and recycled materials showed comparable values over a temperature range from 20 to 100 °C. In addition, the crossover temperature (*T*_cross_), where *G*′ and *G*″ cross,^66,67^ was comparable for pristine (78 ± 2 °C) and recycled (74 ± 2 °C) materials. Frequency sweep experiments (Figure 11) also showed that both pristine and recycled materials reached a constant plateau in *G*′ of around 0.5 MPa, indicating that both materials showed a constant cross-linking density at elevated temperatures, even above the *T*_cross_.

The results from the dissolution-based recycling show that the polyimine CANs can be recycled efficiently without a significant loss in mechanical properties. However, thermomechanical reprocessing of CANs is in most cases still preferred as it generally requires less effort and prevents the use of (large volumes of) solvent. In cases where the requirement of high temperatures causes problems to the materials, however, recycling via dissolution can offer a way out.^68^ Alternatively, “wetting” of the materials can be applied to increase the efficiency of vitrimeric welding.^69^ This wetting can serve as an energy-efficient method to lower the amount of required energy for thermal reprocessing and can be a more sustainable alternative, especially when low-toxicity and greener solvents (e.g., bioethanol) can be used. The varying solubility of specific CANs can also prove useful in solubility-based separation processes of different plastics and other contaminants in waste streams. Additionally, dissolution-based recycling may prove a suitable method for the recovery of the polymer matrix of composite materials.

For future applications, we would last like to briefly discuss the potential of postsynthetic modifications to CANs while in either the dissolved or swollen state. In previous work, we observed that phase separation in polyimine CANs could be reverted by immersing the materials in a good solvent, to which additional monomers could be added and incorporated within the polymer network.^30^ In another example, metal coordination of the dynamic covalent imines could be performed via dissolution of polymer networks.^33^ A study by Zhu and co-workers also revealed a solvent-responsive reversible and controllable conversion between an amorphous network and molecular cage structures.^70^ From the perspective of durability, it can be more feasible to enhance or alter the material properties of old, weak, or damaged materials, rather than making an entirely new material while discarding the old one. However, because many applications still require materials with high solvent resistance, tuning CANs to only (selectively) dissolve in a specific solvent while keeping high resistance toward other solvents may be required.

## Conclusions

Cross-linked polymer networks are essentially considered insoluble in organic solvents. When CANs are considered, however, the issue regarding solubility becomes more complicated. Although dissociative CANs can be easily dissolved when dissociating the network, associative CANs were long thought to be insoluble. However, bond exchange reactions within a CAN, whether proceeding via an associative or dissociative mechanism, enable the material to swell and rupture to eventually rearrange into smaller soluble particles. Depending on the chemical composition of the polymer network and the dynamics of the bond exchange reaction, the penetration of solvent molecules into the polymer network and splitting into soluble parts can be either suppressed or stimulated. In addition, modifications to the network structure were found to affect the size of dissolved polymer particles. We observed that higher cross-linked materials formed larger (but still soluble) particles. We also observed that the size decreased when the concentration of polymer was reduced (i.e., a higher solvent/polymer ratio). Although good solvent resistance might be required for some applications, the (selective) solubility of CANs can also be used advantageously, for example, in chemical recycling or modification of the materials. We envision that our results on imine-based CANs can be easily applied to other CANs, whether they rely on dissociative or associative bond exchange.

## Supporting Information Available

Experimental details of synthesis and analysis, additional NMR and IR spectra, kinetic studies, rheological characterization, additional time-dependent dissolution studies, and protocols for the reversible dissociation study and the recycling study (PDF)
