# Supplementary material for: Probing the Solubility of Imine-Based Covalent Adaptable Networks
Source: ACS Appl Polym Mater. 2023 Dec 19;6(1):79–89. doi: 10.1021/acsapm.3c01472 (PMC10788871; doi:10.1021/acsapm.3c01472)
Supplement: Supplementary file 1 — ap3c01472_si_001.pdf [file ap3c01472_si_001.pdf]

Supporting Information for:

Probing the Solubility of Imine-based Covalent Adaptable Networks

By

Sybren Klaas Schoustra, Vahid Asadi and Maarten Marinus Johannes Smulders*

Laboratory of Organic Chemistry, Wageningen University, Stippeneng 4, 6708 WE Wageningen, The Netherlands.

E-mail: maarten.smulders@wur.nl

Table of content

Overview of used chemicals S3

Specification of used equipment S3

Synthesis of polyimine networks........ S4

Synthesis of V-Urea networks S5

NMR spectra of dissolved polyimines S5

FT-IR spectra........ S9

Reversible dissociation of dissolved polyimines by addition of acid S11

Scatter plots for the solubility of PI-30 as a function of solvent polarity and dielectric constant S12

Imine bond exchange kinetic study S13

Monitoring of the dissolved fraction over time........ S15

Rheological properties of synthesized polyimine CANs........ S16

Differential scanning calorimetry (DSC) S18

Recycling *via* dissolution........ S19

References S20

Overview of used chemicals

An overview of all used chemicals is provided in Table S1, including full names, abbreviations, purity and supplier, and were all used as received.

Table S1. Overview of all used chemicals, including full names, abbreviations, purity and supplier.

Material	Abbreviation	Purity	Supplier
Acetonitrile	MeCN	99.8%	Biosolve
Cadaverine / 1,5-diaminopentane	Cad	98%	TCI
Chloroform	$CHCl_3$	99.8%	Alpha Aesar
1,3-cyclohexanebis(methylamine), mixture of isomers	Cy	98%	Merck
1,5-diamino-2-methylpentane	MeP	99%	Merck
Diethylenetriamine	DETA	99%	Merck
Diethyl ether	Et_2O	99.8%	Honeywell
Dimethyl sulphoxide	DMSO	99.7%	Merck
Ethyl acetate	EtOAc	99.9%	Biosolve
Heptane	Hept	99.%	VWR
Methanol	MeOH	99.8%	Biosolve
Poly(ethylene glycol), bis(3-aminopropyl) terminated. M_n ~1500	$H_2NPr(PEG)PrNH_2$		Merck
2-Propanol	$^{i}PrOH$	99.8%	Biosolve
Terephthalaldehyde	TA	99%	Merck
Tetrahydrofuran	THF	99.8%	Biosolve
4,7,10-Trioxa-1,13-tridecanediamine	TOTDDA	97%	Merck
Tris(2-aminoethyl)amine	TREN	98%	TCI
m-Xylylenediamine	Xyl	99%	Fisher
1,2-Bis(2-aminoethoxy)ethane	DA	98%	TCI

Specification of used equipment

NMR spectra were recorded on a Bruker Avance III 400 MHz instrument and analyzed with MestreNova software. Chemical shifts are reported in parts per million (ppm), calibrated on the residual peak of the solvent, whose values are referred to tetramethylsilane (TMS, δ_{TMS} = 0 ppm), as the internal standard. IR analyses were performed on a Bruker Tensor 27 spectrometer with platinum ATR accessory. Rheological measurements were conducted

on an MCR 301 Anton Paar rheometer in combination with a temperature-controlled plate-plate configuration and temperature controller hood for additional thermal homogeneity.

Synthesis of polyimine networks

All polyimine networks were prepared, according to our previously documented synthesis for polyimine CANs,[S1] by dissolving terephthalaldehyde (**TA**, 4.00 mmol), tris(2-aminoethyl)amine (**TREN**, 0.80 mmol) and either of the specified diamines (2.80 mmol) in a small amount of THF (typically ~5 mL per gram material). The solution was briefly mixed until a homogenous mixture was obtained. It was then poured into a glass petri dish, which was left for overnight. Most of the solvent evaporated to air, and a wet polymer film resulted. The film was further dried in a vacuum oven at 50 °C for at least one day to remove any remaining solvent and water. The formation of the polyimine networks was confirmed by FT-IR analysis when the C=O stretch signal of the aldehyde around 1670 cm^{-1} could no longer be observed and a new imine signal around 1640 cm^{-1} appeared. All polymer films had a typical yellow-to-orange appearance (see Figure S1), which is common to imine materials.

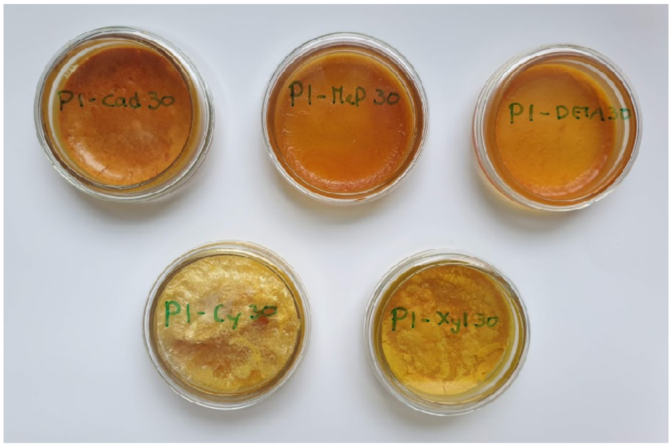

Figure S1. Physical appearance of some polyimine materials, directly after the synthesis, without performance of any post-processing like hot-pressing.

Synthesis of V-Urea networks

The synthesis of V-Urea networks was based on an earlier documented protocol from Du Prez and co-workers.[S2] Ethylenediamine-*N*-*N'*-bis(acetamide) (**EDABA**, 4.00 mmol), **TREN** (0.80 mmol) and either of the specified diamines (3.20 mmol) were mixed together in 10 mL DMF. The mixture was carefully heated with a heat gun, while swirling the flask, to dissolve all material. Then, the mixture was poured into a petri dish, which was placed in an oven at 80 °C for at least 24 hours. The obtained polymers were further dried in a vacuum oven at 50 °C for overnight. The formation of the V-Urea networks was confirmed by FT-IR analysis when the C=O stretch signal of the acetamide around 1700 cm^{-1} could no longer be observed. The V-Urea materials had a yellow-to-orange appearance (see Figure S2).

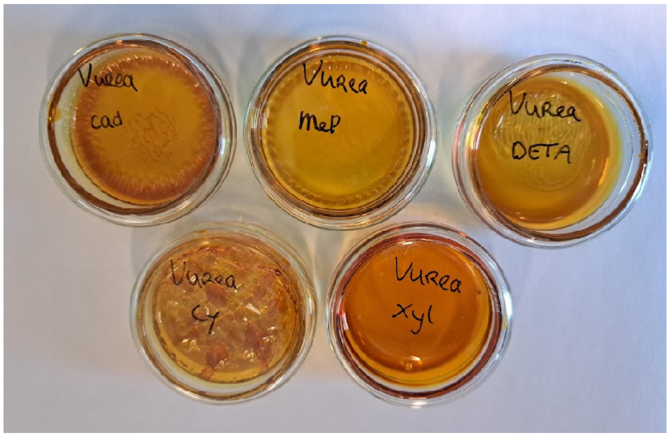

Figure S2. Photo of V-Urea films directly after the synthesis, without performance of any post-processing like hot-pressing.

NMR spectra of dissolved polyimines

The ^1H NMR spectra of the polyimine materials with different diamines are presented in the figures below. Assignment of the characteristic regions are mentioned in the captions under the corresponding figures.

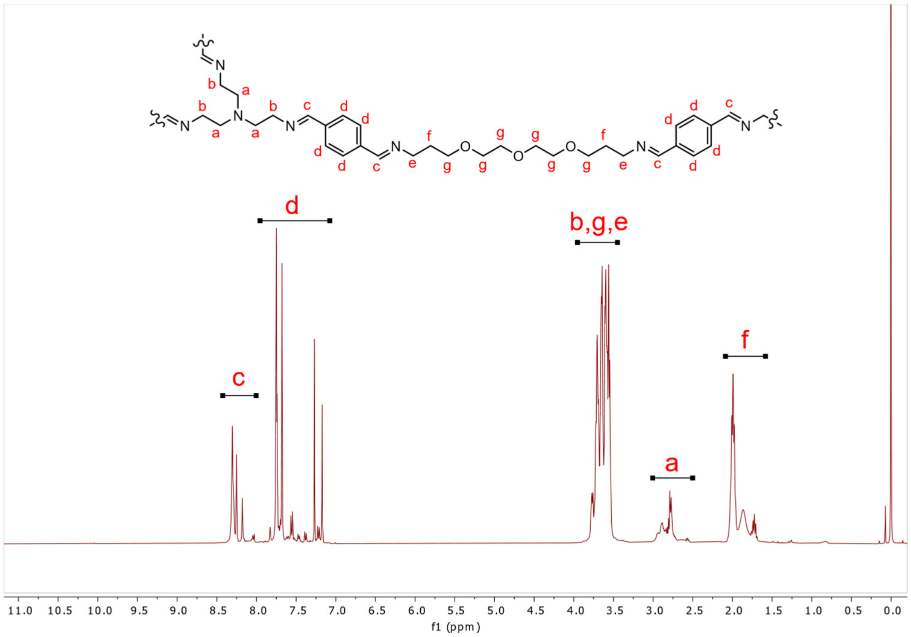

Figure S3. Full ^{1}H NMR spectrum of **PI-30** in $CDCl_3$ (400 MHz, 298 K).

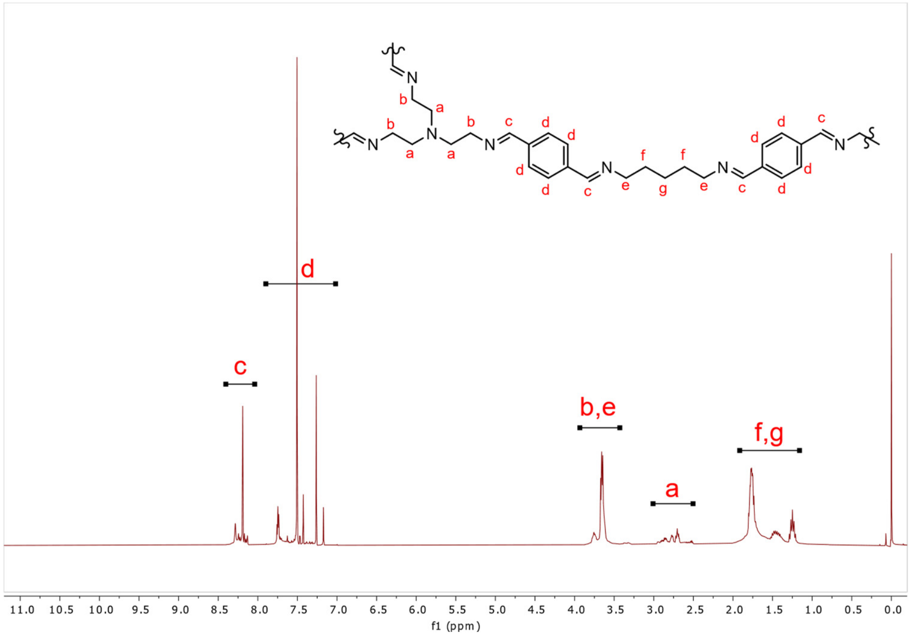

Figure S4. ^{1}H NMR spectrum of **Cad30** in $CDCl_3$ (400 MHz, 298 K).

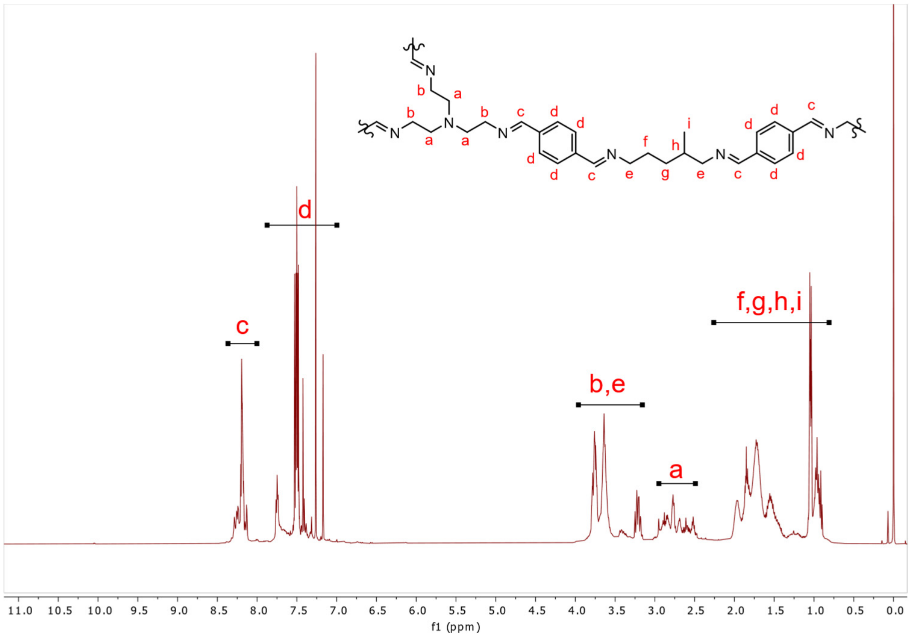

Figure S5. ^{1}H NMR spectrum of **MeP30** in $CDCl_3$ (400 MHz, 298 K).

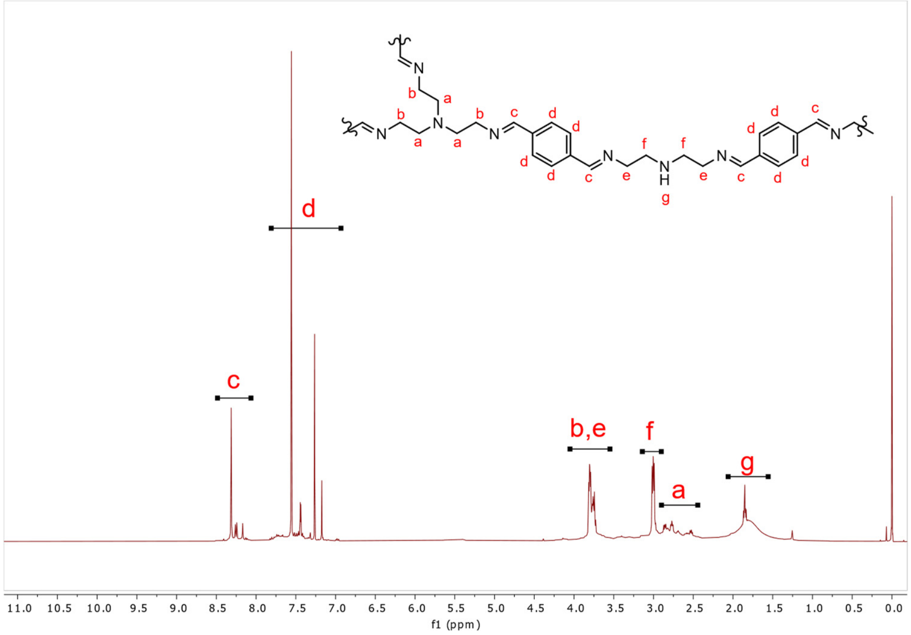

Figure S6. ^{1}H NMR spectrum of **DETA30** in $CDCl_3$ (400 MHz, 298 K).

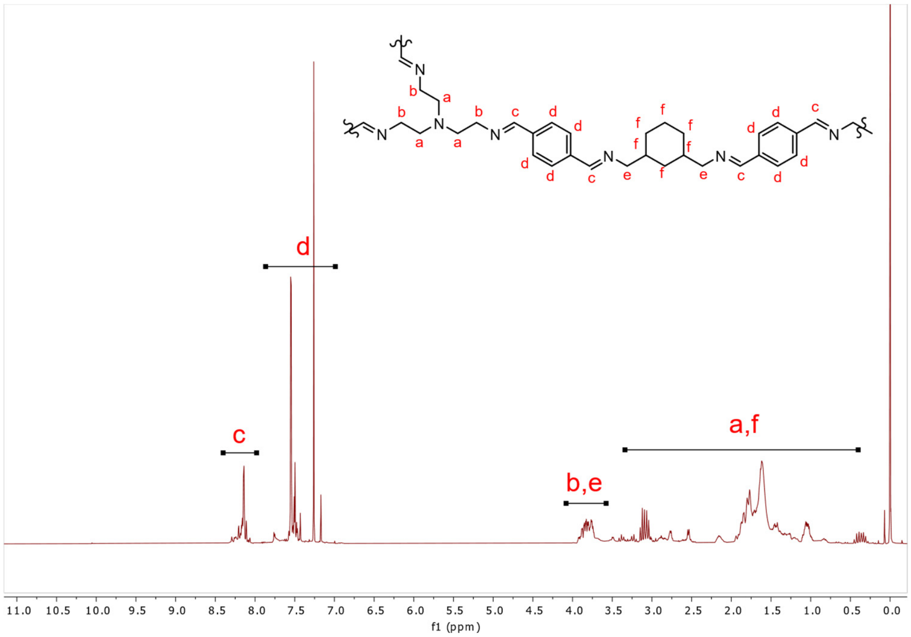

Figure S7. ^{1}H NMR spectrum of **Cy30** in $CDCl_3$ (400 MHz, 298 K).

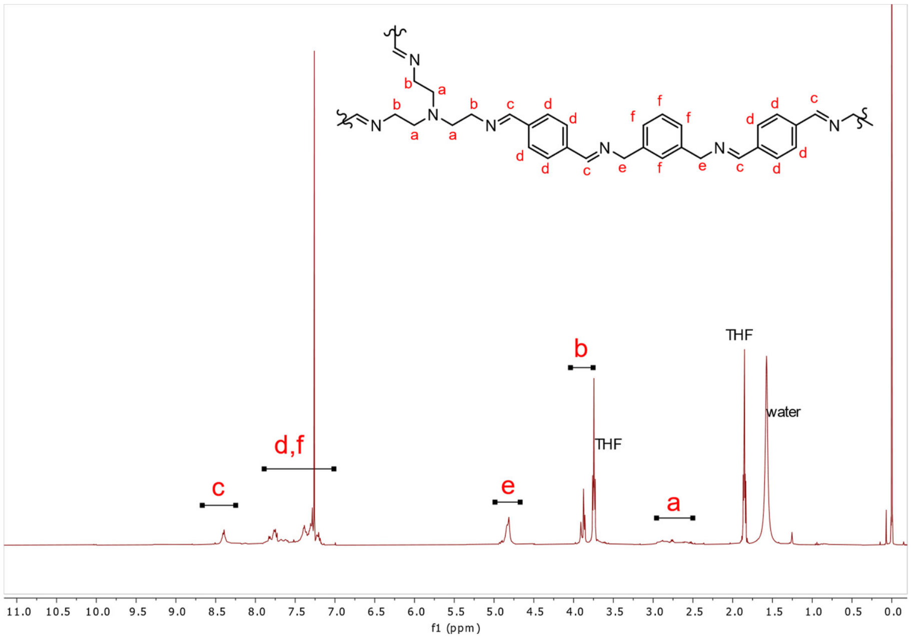

Figure S8. ^{1}H NMR spectrum of **Xyl30** in $CDCl_3$ (400 MHz, 298 K).

FT-IR spectra

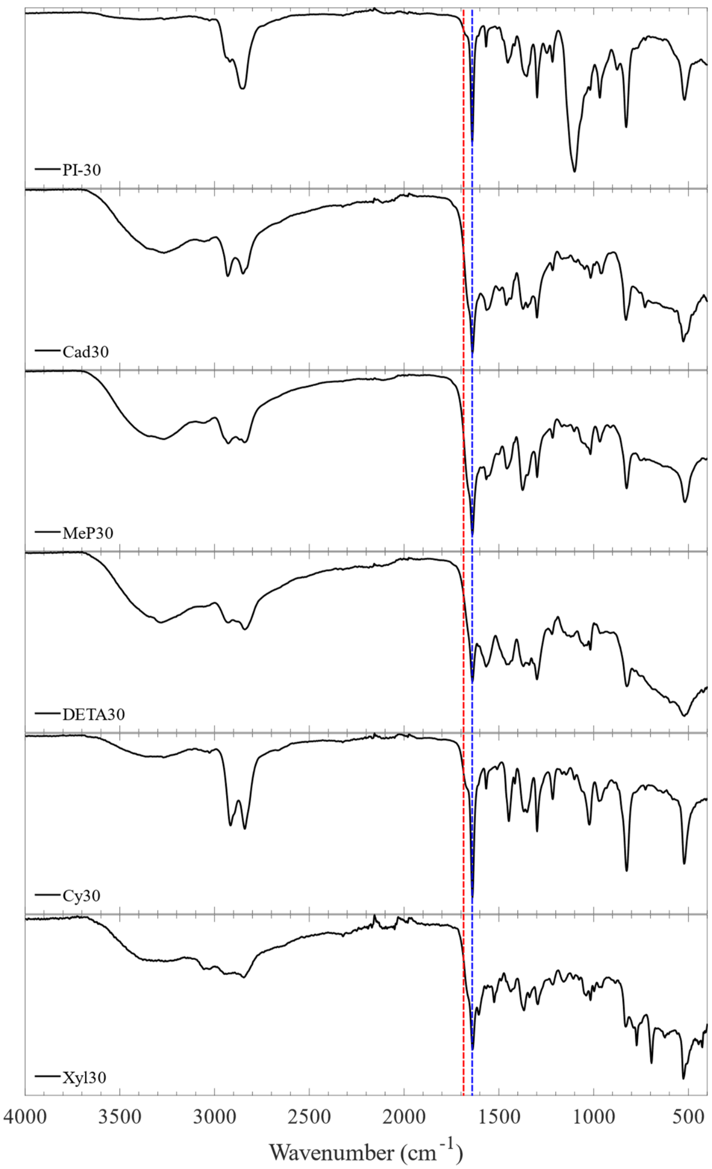

Figure S9. FT-IR spectra for synthesized polyimine CANs. Disappearance of the aldehyde signal (1700 cm^{-1} shown as red dashed line) and appearance of the imine signal (1640 cm^{-1} shown as blue dashed line) was used to confirm formation of the polyimines CANs.

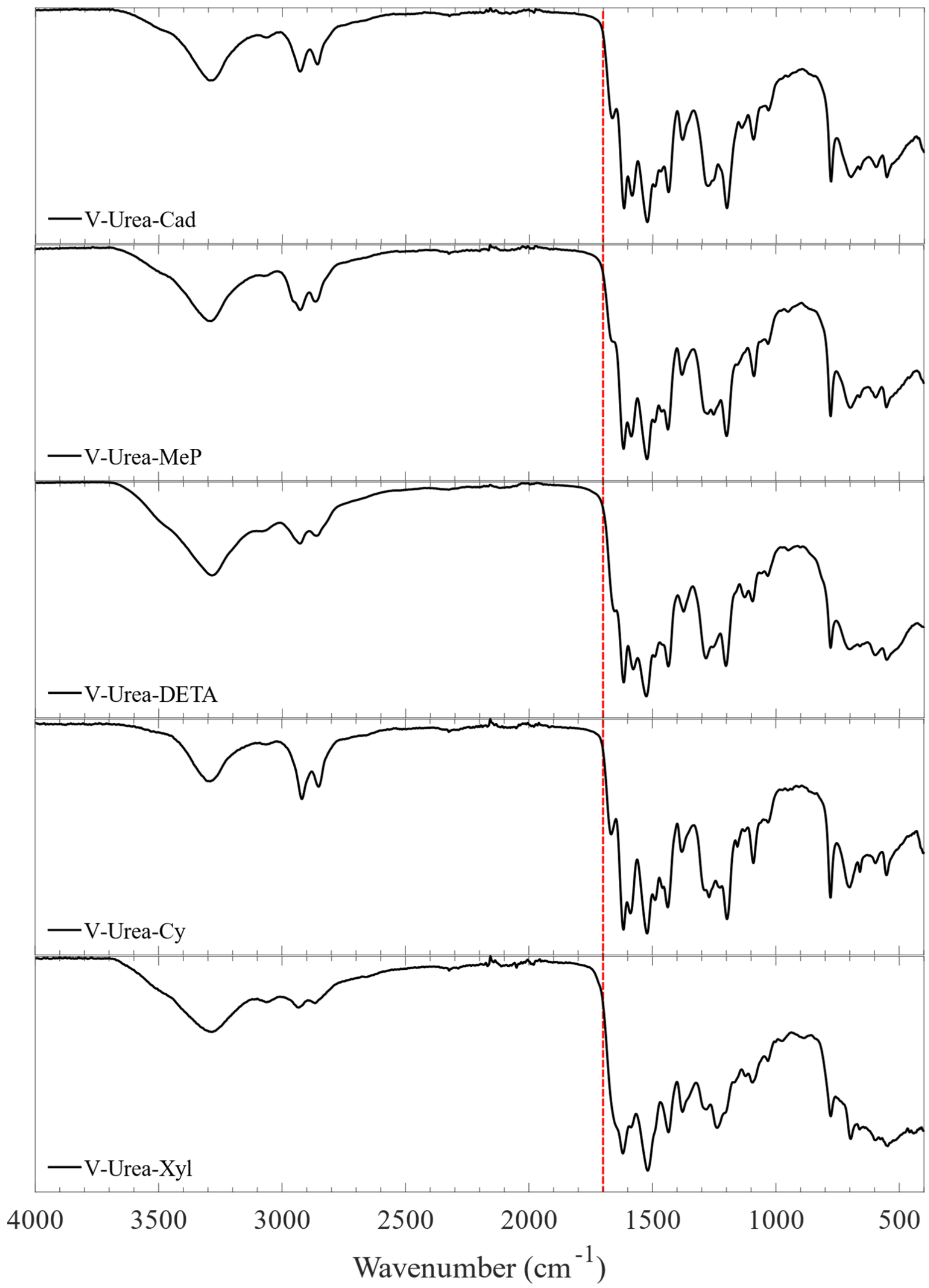

Figure S10. FT-IR spectra for the synthesized V-Urea CANs. Disappearance of the C=O stretch signal of the acetamide ketone around 1700 cm^{-1} (shown in red dashed line) was used to confirm formation of the V-Urea CANs.

Reversible dissociation of dissolved polyimines by addition of acid

The dissociation of imines is known to be catalyzed by acid, and as such we investigated if the polyimines would hydrolyze in more acidic environments. When decreasing the pH to stronger acidic environments we noticed that the imines started to partially dissociate to form a new equilibrium between imine and aldehyde and amine (Figure S11). Solutions were prepared of 0.1 M and 1.0 M acetic acid in 0.5 mL $CDCl_3$, to which 5 mg of **PI-30** was added. ^1H NMR analysis showed that an equilibrium between imines and hydrolyzed products formed instantly, where a higher concentration of acid resulted in the equilibrium being shifted more towards the dissociated products. These results show that in acidic conditions the imines do not fully dissociate, but instead the equilibrium between dissociation and formation of imines is shifted. We then also investigated if we could push the equilibrium back towards formation of the imines. For this, an equimolar amount of the base triethylamine was added, and indeed the equilibrium was rapidly pushed back towards formation of the imines, as only trace amounts (<1%) of aldehyde remained.

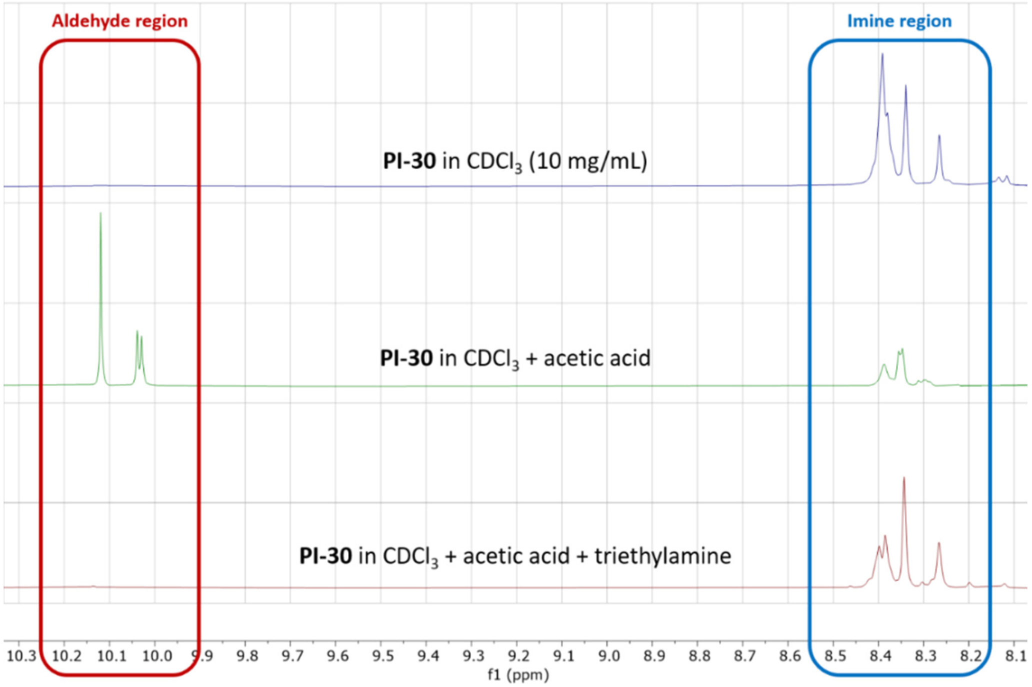

Figure S11. ^1H NMR spectra of **PI-30** in $CDCl_3$ (top), after addition of acetic acid (middle) and when neutralized with triethylamine (bottom). The spectra indicate that after addition of the acid the imines partially dissociate back to aldehyde, but after neutralization with triethylamine the equilibrium is again pushed fully towards imine formation.

Scatter plots for the solubility of PI-30 as a function of solvent polarity and dielectric constant

The dissolved fractions for **PI-30** in several common solvents was plotted as a function of the polarity (Figure S12) and dielectric constant (Figure S13) of the corresponding solvents in the figures below.

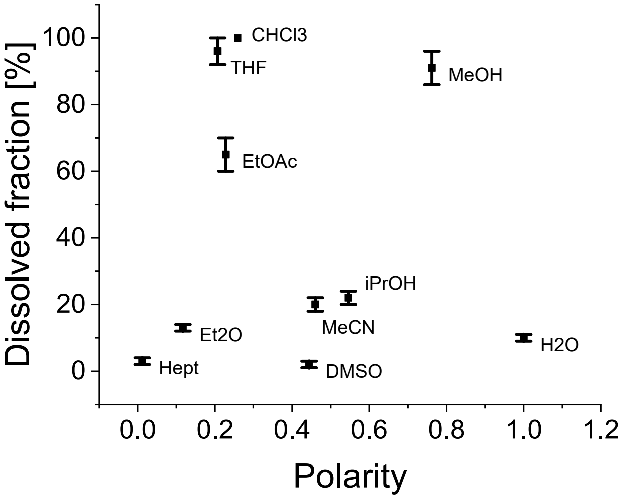

Figure S12. Scatter plot of the dissolved fraction of **PI-30** as a function of solvent polarity. The polarity values have been taken from literature.[S3]

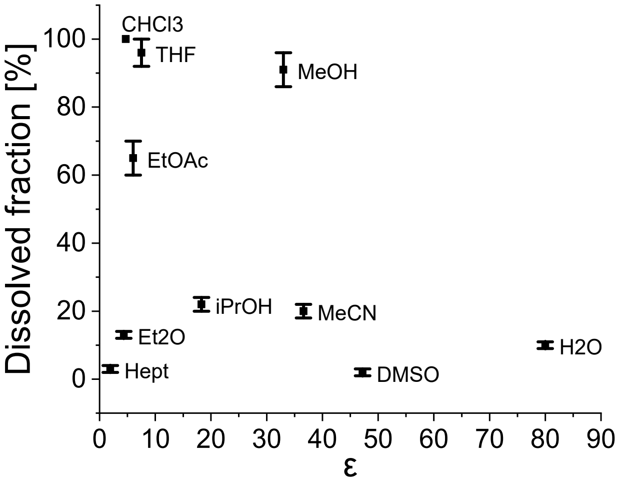

Figure S13 Scatter plot of the dissolved fraction of **PI-30** as a function of the dielectric constant of the solvent.

Imine bond exchange kinetic study

To study the kinetic of imine bond exchange reactions, first (*E*)-*N*-benzyl-1-phenylmethanimine (**BI**), (*E*)-*N*-benzyl-1-(p-tolyl)methanimine (**TI**) and diamine (**DI**) were synthesized based on our previously reported paper.[S4] For the transimination reaction between imine and amine, **BI** with initial concentration of $[BI]_0$ = 14 mM and excess amount (imine:amine 1:30) of a commercially available diamine (**DA**) (structurally similar to the diamine used in the synthesis of polyimine networks) were dissolved in three different deuterated solvents (chloroform, DMSO, and acetonitrile) and followed by ^{1}H NMR spectroscopy at 25 °C, as shown in Figure S14.

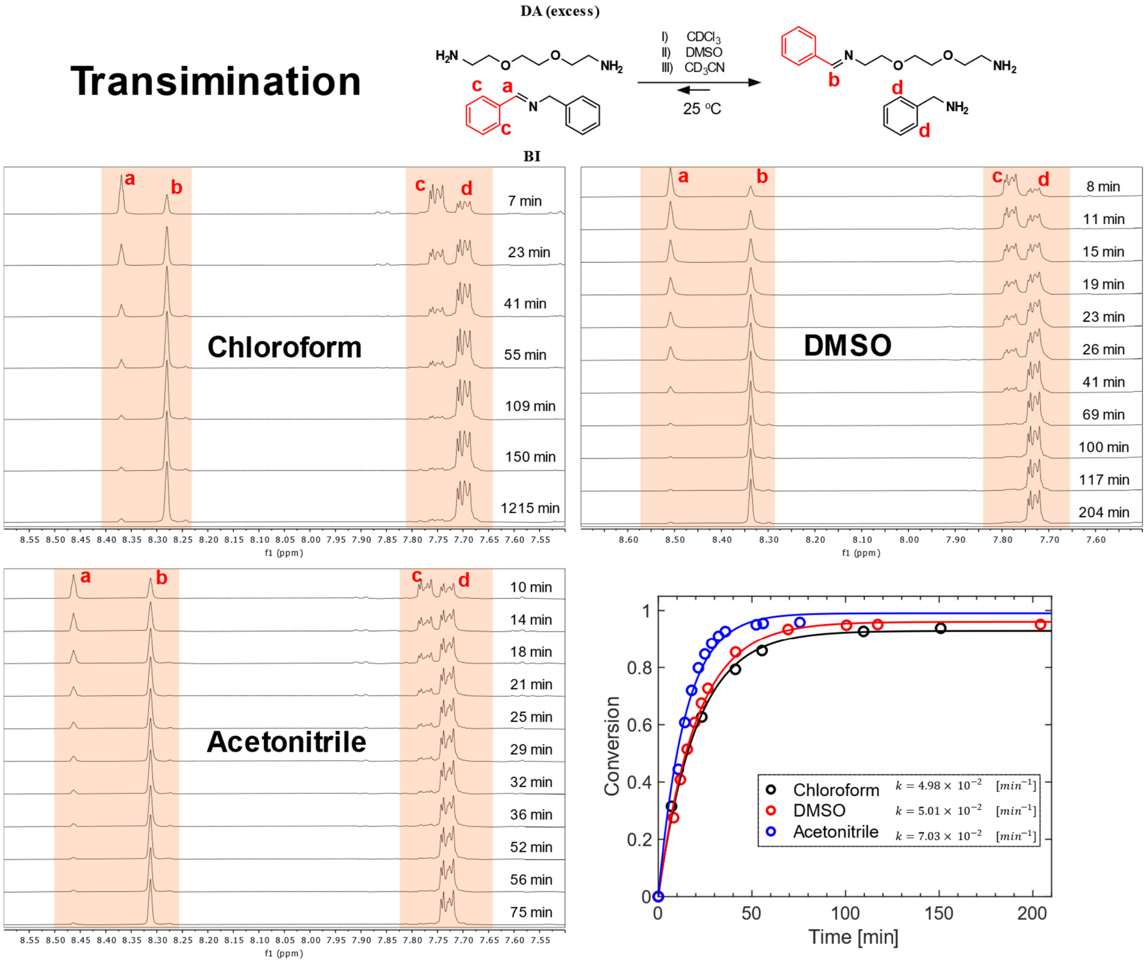

Figure S14. Stacked ^{1}H NMR spectra of the transimination reaction with excess amount of **DA** as nucleophile in three different solvents at 25 °C, zoomed in the imine region (8.6 – 7.5 ppm). The transimination reaction (conversion) is followed over time by integration of imine signals of the corresponding materials (shown as protons "a" and "b" in the reaction scheme). Another peak that can be used to calculate the conversion is the ring protons of **BI** and newly formed benzylamine (shown as protons "c" and "d" in the scheme). The kinetic plots

of the conversion over time in different solvents are presented in the bottom-right panel. Due to addition of the excess of nucleophilic reactant (**DA**), a *pseudo*-first order reaction could be assumed. The data was therefore fitted with the model for first-order kinetics to derive the rate constants listed inside the plot.

For the imine metathesis reaction between two different imines, **TI** with initial concentration of $[BI]_0$ = 15 mM and excess amount (imine:imine 1:30) of synthesized diimine (**DI**) were dissolved in three different deuterated solvent (chloroform, DMSO, and acetonitrile) and followed by ^{1}HNMR spectroscopy at 25 °C as shown in Figure S15.

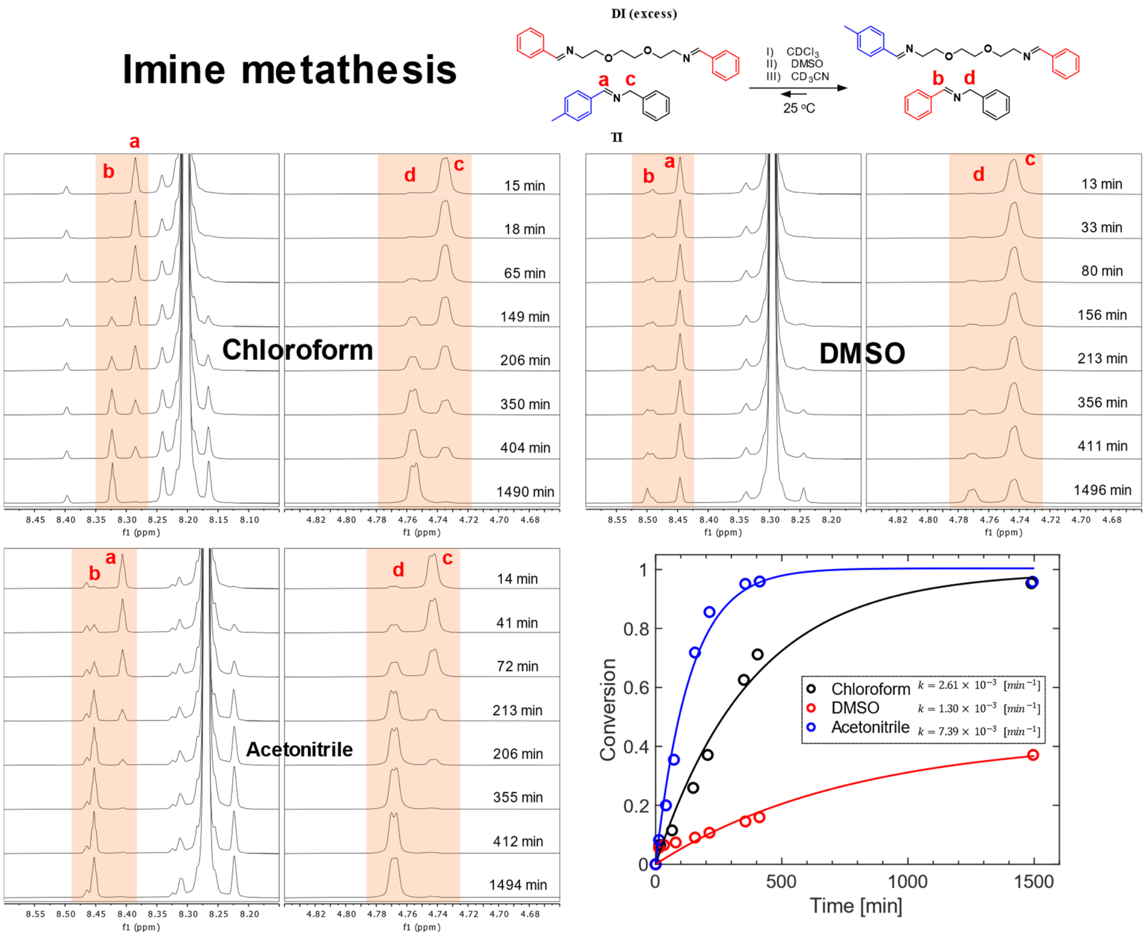

Figure S15. Stacked ^{1}H NMR spectra of the imine metathesis reaction with excess amount of **DI** as nucleophile in three different solvents at 25 °C , zoomed on two different region, *i.e.* imine region (around 8.6 – 8.1 ppm), and the proton next to imine (around 4.83 – 4.67 ppm). The imine metathesis reaction (conversion) is followed over time by integration of proton signals next to imine of the corresponding materials (shown as protons "c" and "d" in the reaction scheme) due to better isolated peaks in this region. Another peak that can be used to calculate the conversion is imine protons of **TI** and newly formed **BI** (shown as protons "a" and "b" in the scheme), however, there are some overlaps in this region. The kinetic plots of the conversion over time in different solvents are presented in the bottom-right panel. Due to addition of the excess of nucleophilic reactant (**DI**), a *pseudo*-first order reaction could be assumed. The data was therefore fitted with the model for first-order kinetics to derive the rate constants listed inside the plot.

Monitoring of the dissolved fraction over time

The dissolution of **PI-30** was monitored over time in three different solvents where 100 mg of sample was added to 10 mL of solvent. The dissolved fraction was determined after separating the liquid and solid phase at different time points and drying the remaining solid phase. A characteristic dissolution onset-time (time needed for 5% weight loss) has been defined as t_0 to compare the solubility strength of different solvents.

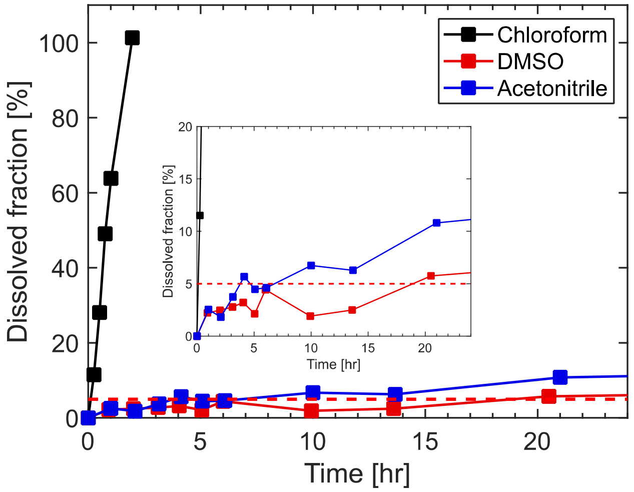

Figure S16. Monitoring of the **PI-30** dissolution over time in three different solvents. The red dashed line represent 5% dissolved fraction which is used to determine the characteristic dissolution onset-time.

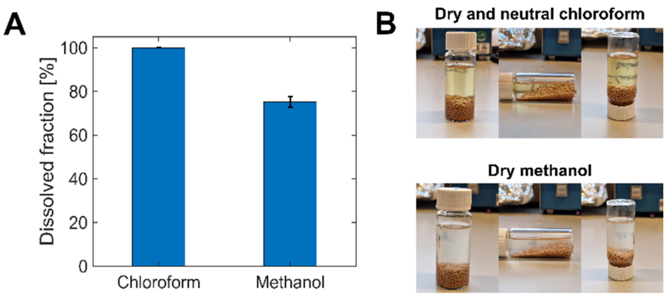

Figure S17. Additional experiments A) Solubility experiment (100 mg per 10 mL of solvent for 10 days) repeated using vigorously anhydrous methanol and neutral and anhydrous chloroform, ruling out the potential effect of trace amount of water or acid (HCl in chloroform). B) Monomers with accounting the same concentration (100 mg/10 mL) were dissolved in the solvents with molecular sieves to remove water during imine formation, showing that the solution does not gel. For this experiment, **TREN** (31.3 mg, 0.14 mmol), **TOTDDA** (66.7 mg, 0.50 mmol), and **TA** (103.8 mg, 0.71 mmol) were dissolved in 20 mL of solvent.

Rheological properties of synthesized polyimine CANs

Oscillatory temperature sweep experiments were performed using parallel plate geometry on polyimine sample disks of 10 mm in diameter and the gap size of 0.650 mm with 0.1% strain at two different frequencies of 1 Hz and 10 Hz. A temperature ramp was applied by slowly heating the materials from 20 to 150 °C, at a heating rate of 1 °C/min. *G*′, *G*′′ and tan(δ) were plotted as a function of the temperature. For the same samples, oscillatory frequency sweep experiments at 100 and 130 °C were performed with a strain of 0.1%, while the frequency was increased logarithmically from 0.1 till 100 Hz. *G*′ and *G*′′ were then plotted as a function of the frequency. These results are shown in **Figure S18.**

Sample **Xyl30** showed an increase in modulus starting around 110 °C which was also reproducible. To investigate the underlying reason of this increase, we repeated the temperature sweep at 0.1 strain and 10 Hz with low heating rate of 0.5 °C/min, shown in Figure S19. When the sample is heated very slowly, it has more time to equilibrate and the sharp increase in the modulus disappears. One possible explanation for this trend is that at a certain temperature around 120 °C the mobility of chain significantly increases (supported by the DSC data which is shown in the next section), allowing the aromatic units to be aligned and establish π–π stacking which leads to higher modulus. However, by reducing the heating rate and give more time to the sample to equilibrate, the rearrangement and π–π stacking happen more gradually.

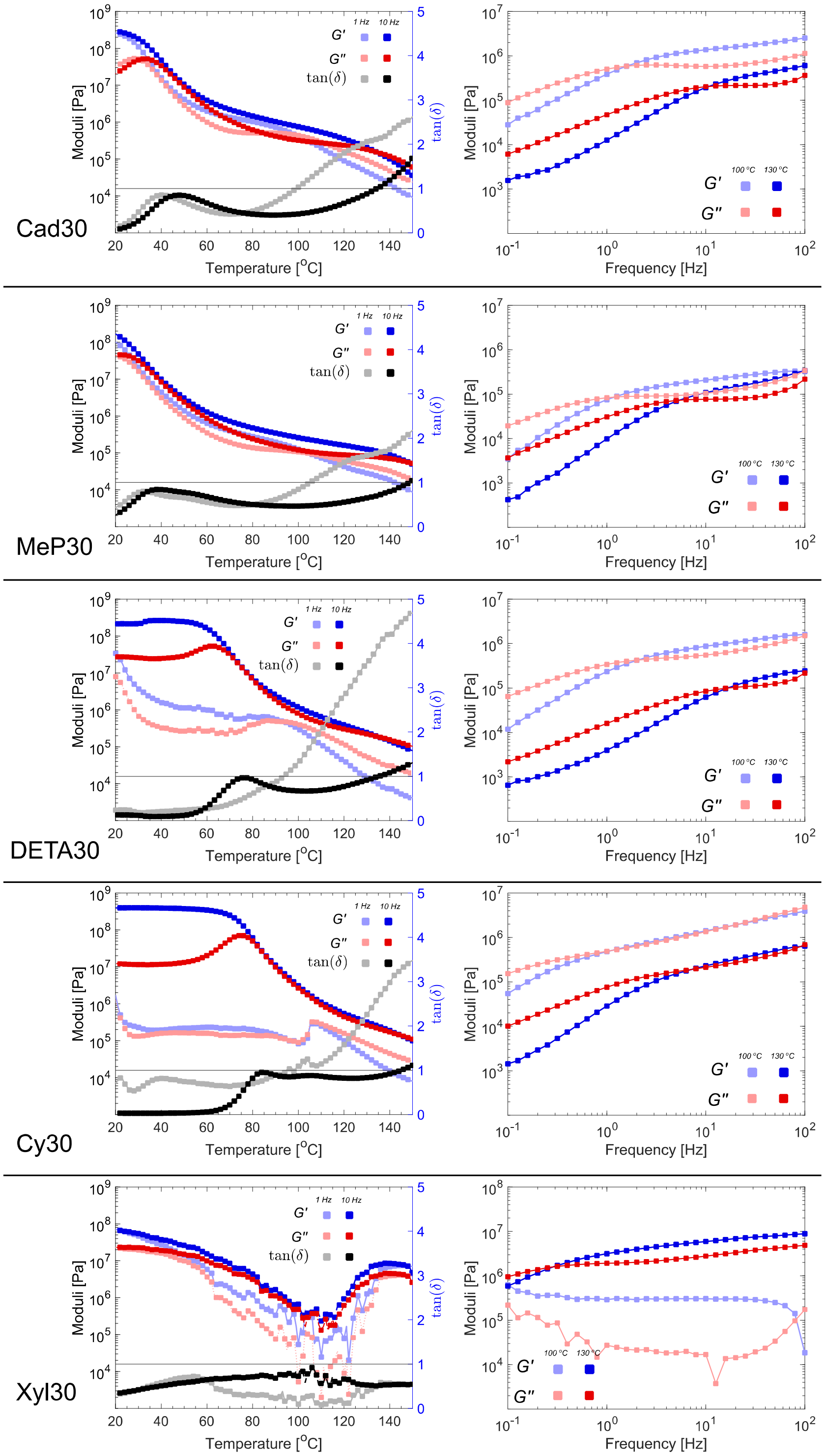

Figure S18. Temperature sweep (left panels) and frequency sweep (right panels) measurements for the five different polyimine CANs.

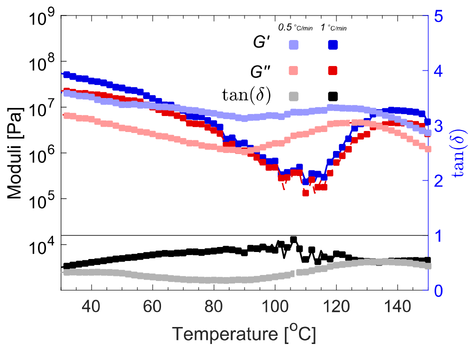

Figure S19. Temperature sweep measurement for the sample **Xyl30** with two different heating rate of 0.5 and 1 °C/min, at 0.1 strain and 10 Hz.

Differential scanning calorimetry (DSC)

Differential scanning calorimetry was performed following ISO 11357 using a Perkin Elmer DSC 8000 which was cooled by a liquid nitrogen cooling system. Large volume (60 μL) stainless steel cups were used to hold the sample and used as a reference. The sample first equilibrated at −90 °C for 10 min and then heated to 150 °C with a heating rate of 10 °C/min, followed by a fast cooling (100 °C/min) to −90 °C. After equilibrating at −90 °C for 10 min, the second heating was performed again to 150 °C with the rate of 10 °C/min. From the graph a T_g of 56 °C and melting point of 125 °C were observed for the sample **Xyl30**. These results also support out explanation regarding modulus increase in the rheological measurements and rule out that the modulus increase does not originate from crystallization.

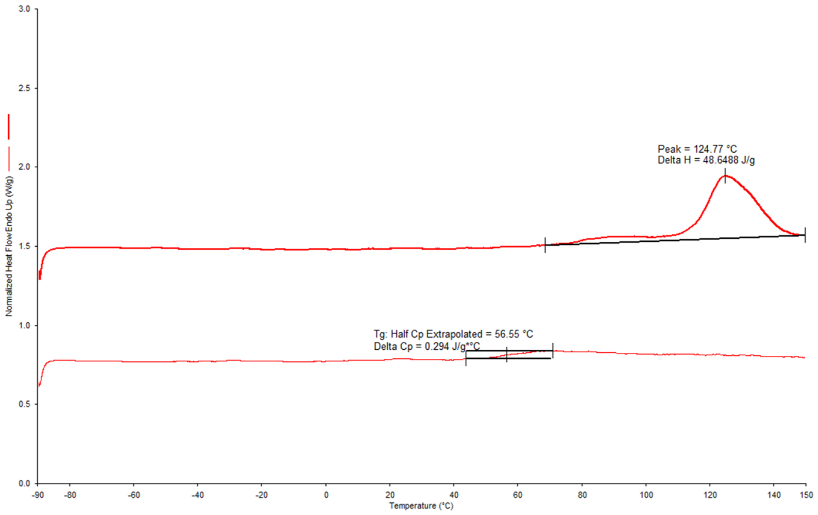

Figure S20. DSC curve of sample **Xyl30** showing the first (top) and second (bottom) heating run after a fast cooling step in the range of −90 to 150 °C.

Recycling *via* dissolution

For the recycling experiment, a pristine **PI-30** material was first synthesized.

A temperature sweep experiment was performed to determine the T_{cross} (where tan(δ) = 1),[S5] giving the temperature at which the material transitions from a rubbery to a viscous (malleable) state.[S6] The materials were analyzed as discs with a 10 mm thickness and 1.0 mm thickness. Using a rheometer, the samples were oscillated with 0.1% strain at a frequency of 1 Hz. A temperature ramp was applied by heating the materials from 20 to 100 °C, at a heating rate of 1 °C per 20 s. G', G'' and tan(δ) were plotted as a function of the temperature.

Next, frequency sweep experiments at several temperatures were performed to check for a constant plateau of the G', indicating that the crosslinking density remains constant at elevated temperatures.[S7-S9] For this, polymer discs with a 10 mm diameter and 1.0 mm thickness were again used. Using a rheometer, the samples were oscillated with a strain of 0.1%, while the frequency was increased logarithmically from 0.1 till 100 Hz. G' and G'' were then plotted as a function of the frequency. The experiment was repeated at 40, 60, 80 and 100 °C.

After the pristine materials were fully analyzed, they were fully dissolved in THF. The solution was then poured into a petri and the solvent was slowly evaporated to air overnight. The newly obtained polymer film was further dried in a vacuum oven at 50 °C for one day. The same rheology tests as for the pristine materials were performed to compare the material properties of the pristine material to the recycled material.

Below, photos are presented of a pristine and recycled polymer film of **PI-30**.

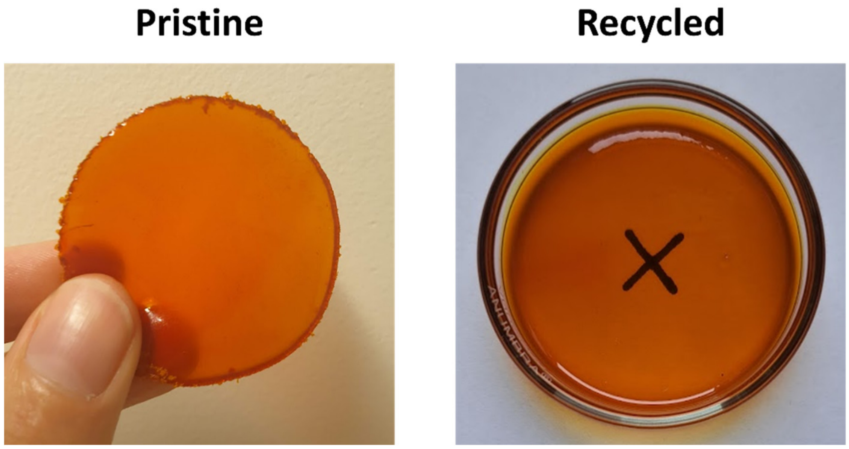

Figure S21. Photos of pristine and recycled polymer films of **PI-30**. The black cross was drawn on the white paper below the petri dish to indicate the transparency of the polymer film.

References

(S1) Schoustra, S. K.; Dijksman, J. A.; Zuilhof, H.; Smulders, M. M. J. Molecular control over vitrimer-like mechanics – tuneable dynamic motifs based on the Hammett equation in polyimine materials. *Chem. Sci.*, **2021**, *12*, 293–302.

(S2) Denissen, W.; De Baere, I.; Van Paepegem, W.; Leibler, L. ; Winne, J.; Du Prez, F. E. Vinylogous Urea Vitrimers and Their Application in Fiber Reinforced Composites. *Macromolecules*, **2018**, *51*, 2054–2064.

(S3) Reichardt, C. and Welton, T. Appendix A. Properties, Purification, and Use of Organic Solvents. Solvents and Solvent Effects in Organic Chemistry, **2010**, 549-586, Wiley-VCH Verlag GmbH & Co.

(S4) Schoustra, S. K.; Groeneveld, T.; Smulders, M. M. J. The effect of polarity on the molecular exchange dynamics in imine-based covalent adaptable networks. *Polym. Chem.,* **2021**, *12*, 1635–1642.

(S5) Adzima, B. J.; Aguirre, H. A.; Kloxin, C. J.; Scott T. F.; Bowman, C. N. Rheological and Chemical Analysis of Reverse Gelation in a Covalently Cross-Linked Diels−Alder Polymer Network. *Macromolecules*, **2008**, *41*, 9112–9117.

(S6) Liu, W.; Yang, S.; Huang, L.; Xu J.; Zhao, N. Dynamic covalent polymers enabled by reversible isocyanate chemistry. *Chem. Commun.* **2022**, *58*, 12399–12417.

(S7) Montarnal, D.; Capelot, M.; Tournilhac, F.; Leibler, L. Silica-Like Malleable Materials from Permanent Organic Networks. *Science*, **2011**, *334*, 965–968.

(S8) Capelot, M.; Unterlass, M. M.; Tournilhac, F.; Leibler, L. Catalytic Control of the Vitrimer Glass Transition. *ACS Macro Lett.*, **2012**, *1*, 789–792.

(S9) Capelot, M.; Montarnal, D.; Tournilhac, F.; Leibler, L. Metal-Catalyzed Transesterification for Healing and Assembling of Thermosets. *J. Am. Chem. Soc.*, **2012**, *134*, 7664–7667.
